# Association between adenomyosis and maternal and neonatal outcomes: a systematic review and meta-analysis

**DOI:** 10.3389/fmed.2026.1772838

**Published:** 2026-03-31

**Authors:** Yi Liao, Linxi Li, Yuanjian Tong, Yanyan Zhou

**Affiliations:** 1 Department of Obstetrics and Gynecology, Chengdu Shuangnan Hospital, Chengdu, China; 2 Department of Clinical Medicine, North Sichuan Medical College, Nanchong, China; 3 Department of Obstetrics and Gynecology, The People’s Hospital of Renshou County, Meishan, China

**Keywords:** adenomyosis, assisted reproductive technology, live birth, meta-analysis, miscarriage, pregnancy outcomes, preterm birth

## Abstract

**Background:**

Adenomyosis has garnered increasing attention for its association with adverse pregnancy outcomes in recent years. However, findings across studies remain inconsistent. This meta-analysis of cohort studies evaluated the impact of adenomyosis on maternal and perinatal outcomes.

**Methods:**

A systematic search of PubMed, Embase, Web of Science, and the Cochrane Library was conducted to identify relevant studies published from their inception to September 25, 2025. Eligible studies included those comparing pregnancy and obstetric outcomes between women with adenomyosis and those without. To quantify the association between adenomyosis and unfavorable outcomes, meta-analyses were conducted using R 4.3.2 and STATA 12.0, yielding risk ratios (RRs) and 95% confidence intervals (CIs). Heterogeneity across studies was evaluated using Cochran’s Q test, I^2^ statistics, and 95% prediction intervals (PIs).

**Results:**

This meta-analysis synthesized data from 25 cohort studies, encompassing 24,113 women diagnosed with adenomyosis and 8,890,014 controls. The pooled analysis revealed that compared with controls, patients with adenomyosis exhibited significantly lower clinical pregnancy rate (RR [95% CI] = 0.753 [0.625–0.907], 95% PI: 0.433–1.311) and live birth rate (RR [95% CI] = 0.613 [0.469–0.801], 95% PI: 0.254–1.479), alongside markedly higher risks of miscarriage and preterm birth (all *p* < 0.05). The analysis also identified an increased risk of adverse outcomes, including small for gestational age, placenta previa, cesarean section, low birth weight (LBW), pre-eclampsia, postpartum hemorrhage, hypertensive disorders of pregnancy, and preterm premature rupture of membranes among patients with adenomyosis (all *p* < 0.05). Subgroup analyses indicated that women undergoing assisted reproductive technology continued to experience diminished clinical pregnancy and live birth rates, as well as increased risks of miscarriage, placenta previa, LBW, and pre-eclampsia. Moreover, the findings suggested that Asian populations may be more susceptible to pregnancy complications and adverse obstetric outcomes compared to Caucasian populations.

**Conclusion:**

Adenomyosis is associated with various adverse pregnancy and neonatal outcomes. Pregnancies affected by this condition should be classified as high-risk, necessitating careful medical supervision and prompt, tailored interventions to mitigate potential complications throughout gestation.

## 1 Introduction

Adenomyosis is a gynecological condition characterized by the abnormal presence of endometrial glands and stroma within the deeper layers of the myometrium, accompanied by surrounding tissue thickening and proliferation (1, 2). This disease presents a heterogeneous spectrum in both anatomical and clinical manifestations, ranging from a normal-sized uterus to significant enlargement, and from severe dysmenorrhea and hypermenorrhea to asymptomatic cases (3). It is often found concurrently with endometriosis (4). Traditionally, adenomyosis was thought to primarily affect older women, as it was typically identified through histopathological examination after hysterectomies (5). However, recent findings indicate that transvaginal ultrasound (TVUS) can effectively diagnose adenomyosis in younger individuals as well (5, 6). The implications of adenomyosis on women’s quality of life and reproductive health are profound, with principal symptoms including painful menstruation, irregular uterine bleeding, and chronic pelvic discomfort (7).

The prevalence of adenomyosis among pregnant individuals has risen in recent years, driven by a global trend toward delayed childbearing and advancements in infertility treatments, including assisted reproductive technology (ART) (8). This rise has been accompanied by an increase in pregnancy-related complications linked to adenomyosis. Evidence from earlier studies has identified adenomyosis as a significant risk factor for adverse perinatal outcomes, such as pre-eclampsia, preterm birth, postpartum hemorrhage, small for gestational age (SGA), fetal growth restriction, placental abruption, and preterm premature rupture of membranes (PPROM) (9–11). A recent systematic review further corroborated these associations, reporting negative impacts on fertility, pregnancy, and neonatal health (12). However, inconsistencies across studies remain (13–16), leaving the connection between adenomyosis and pregnancy and obstetric complications unclear (17). Many investigations in this area have been constrained by confounding variables, including the use of ART and coexisting conditions like endometriosis (18), both of which are independently related to poor pregnancy outcomes (17, 19).

With an increasing number of recent studies investigating the association between adenomyosis and pregnancy or obstetric outcomes in women (17, 20–23), there is a pressing need to integrate and analyze the existing data systematically. Unlike previous meta-analyses that primarily assessed the association between adenomyosis and fertility outcomes (24, 25), our study exclusively included cohort studies to evaluate the potential causal relationship between adenomyosis and adverse maternal and neonatal outcomes. Furthermore, subgroup analyses were performed to explore the impact of adenomyosis on pregnancy and obstetric outcomes based on different modes of conception, such as ART and natural conception, as well as across different racial populations. These analyses provide novel insights into how adenomyosis may differentially affect outcomes in specific patient subgroups. The findings aim to provide actionable clinical evidence to guide preconception management and perinatal care in patients with adenomyosis, ultimately reducing the risk of adverse maternal and perinatal outcomes.

## 2 Methods

### 2.1 Study protocol

This meta-analysis was registered with the International Prospective Register of Systematic Reviews (PROSPERO) under the registration code CRD420251250250. The study adhered to the standards outlined in the Preferred Reporting Item for Systematic Reviews and Meta-analysis (PRISMA) guidelines (26).

### 2.2 Search strategy

A comprehensive search of electronic databases, including PubMed, Embase, Web of Science, and the Cochrane Library, was performed to identify relevant studies published up to September 25, 2025, without language restrictions. Two independent researchers conducted the search using a combination of keywords such as (“adenomyosis” and “adenomyoses”) AND (“pregnancy,” “fertility,” “neonatal outcomes,” “obstetric outcomes,” “reproductive outcomes,” “miscarriage,” “clinical pregnancy rate,” “live birth rate,” “implantation rate,” “preterm birth,” “cesarean section,” “low birth weight,” “small for gestational age,” “placenta previa,” “pre-eclampsia,” “hypertensive disorders of pregnancy,” “preterm premature rupture of membranes,” and “postpartum hemorrhage”) AND (“cohort,” “prospective,” “retrospective,” and “longitudinal”). Details of the search strategy are provided in Supplementary Files 1. To ensure thoroughness, reference lists of selected studies and relevant reviews were also screened manually for additional eligible publications.

### 2.3 Inclusion and exclusion criteria

The study selection criteria included: (i) cohort studies; (ii) research that investigated maternal and perinatal outcomes in women diagnosed with adenomyosis; (iii) the control group consisted of women without adenomyosis, with a normal uterus, or with infertility solely due to tubal factors; (iv) studies reporting risk estimates, including odds ratios (ORs) or risk ratios (RRs) with corresponding 95% confidence intervals (CIs), to evaluate the association between adenomyosis and maternal or perinatal outcomes. Studies were excluded if they met any of the following conditions: (i) case–control or cross-sectional designs, or cohort studies lacking a control group; (ii) the population under investigation did not include women with adenomyosis; (iii) the required outcomes were not reported or the data necessary for analysis was unavailable; (iv) animal studies, case reports, conference abstracts, reviews, or letters.

### 2.4 Data extraction

Data from eligible studies were independently extracted by two authors using a pre-designed collection form. The extracted information included details on the first author, publication year, study design, study location, method of adenomyosis diagnosis, selection of control population, sample size and age distribution of participants, subtype of adenomyosis, mode of conception, adjusted confounders, and reported outcomes. The primary outcomes of interest encompassed clinical pregnancy rate, live birth rate, implantation rate, miscarriage, and preterm birth. Secondary outcomes included SGA, placenta previa, cesarean section, low birth weight (LBW), pre-eclampsia, postpartum hemorrhage, hypertensive disorders of pregnancy (HDP), and PPROM. The World Health Organization (WHO) defines preterm birth as delivery occurring before 37 weeks of gestation, while LBW refers to infants weighing less than 2,500 grams. SGA is categorized as newborns with a birth weight below the 10th percentile (27).

### 2.5 Quality assessment

The Newcastle-Ottawa Scale (NOS) for cohort studies was employed to evaluate the methodological quality of the included studies (28). The NOS comprises nine criteria, divided into three domains: selection of the study and control groups (maximum of 4 points, reflecting selection bias), comparability between groups (up to 2 points, addressing confounding bias), and assessment of outcomes or exposures (maximum of 3 points, indicating measurement bias). Based on the total NOS scores, studies were classified as low quality (0–3 points), moderate quality (4–6 points), or high quality (7–9 points) (29).

### 2.6 Statistical analysis

The association between adenomyosis and maternal or perinatal outcomes was evaluated by aggregating RRs or ORs along with their 95% CIs extracted from the included studies. Given the relatively low incidence of most outcomes, we uniformly combined ORs and RRs into overall RR values to more intuitively reflect the impact of adenomyosis on adverse maternal and neonatal outcomes. Variability across study findings was quantified using Cochran’s Q test, I^2^ and Tau^2^ statistics, as well as the 95% prediction interval (PI) (30, 31). Heterogeneity was classified as low to moderate when I^2^ ranged from 0 to 50%, whereas values exceeding 50% indicated substantial heterogeneity. A random-effects model was employed when significant heterogeneity was observed; otherwise, a fixed-effects model was utilized (32). Subgroup analyses were conducted to explore differences based on population ethnicity and mode of conception. Sensitivity analysis was performed by sequentially excluding individual studies to test the robustness of the results. Funnel plots, alongside Begg’s (33) and Egger’s (34) tests, were utilized to detect potential publication bias. Statistical significance was defined as a two-sided *p*-value below 0.05. All statistical analyses were performed using R 4.3.2 and STATA 12.0.

## 3 Results

### 3.1 Study selection results

**Figure 1:**
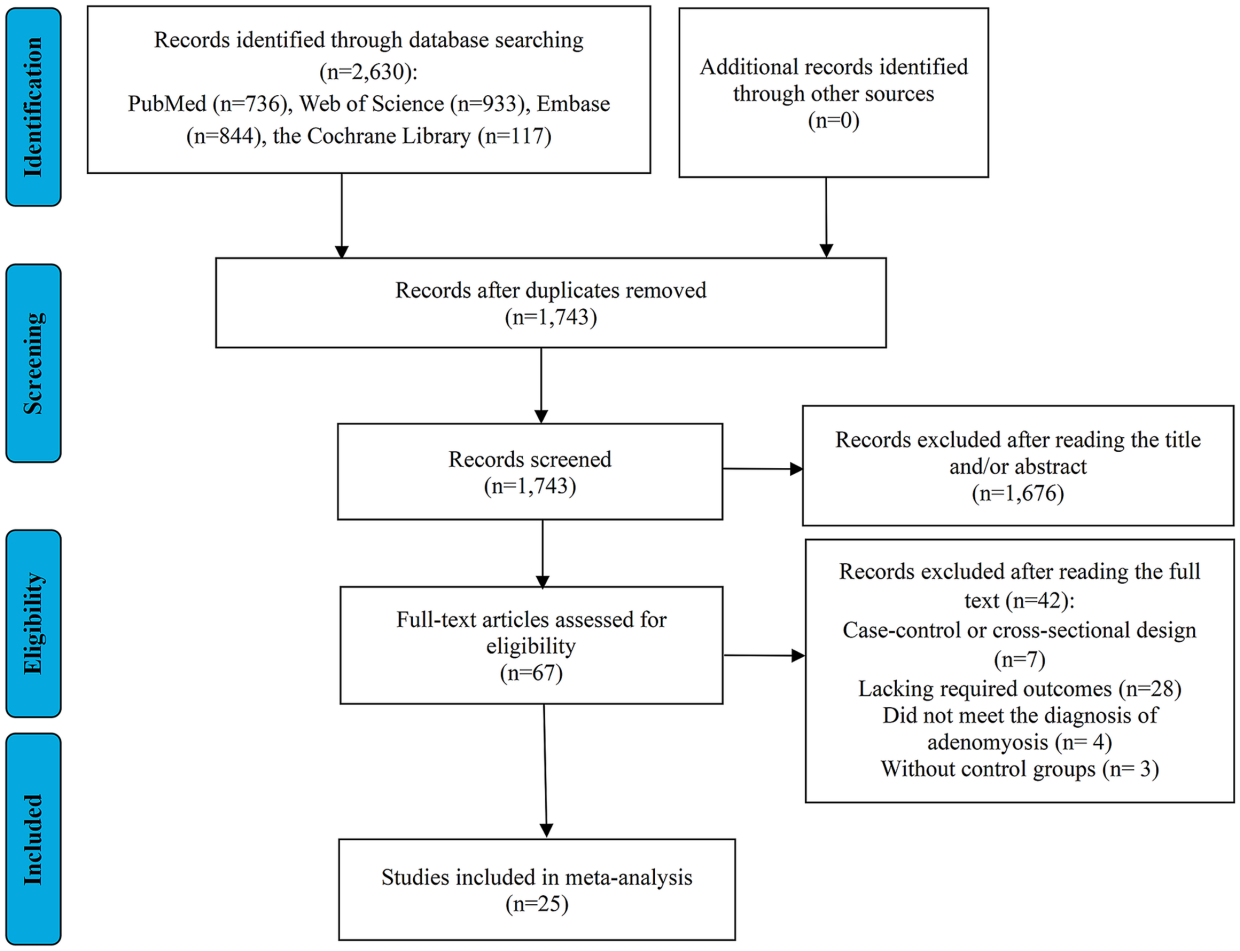
Flow diagram of the process of study selection.

A total of 2,630 records were identified through database searches using the predefined keywords. After removing duplicate entries, 1,743 articles remained for further evaluation. Following an initial screening of titles and abstracts, 1,676 studies were excluded for being unrelated to the research topic. This left 67 articles eligible for full-text assessment. Of these, 7 studies were excluded due to their case–control or cross-sectional design, 28 lacked the necessary RRs or ORs with 95% CIs for maternal or perinatal outcomes, 4 did not meet the diagnostic criteria for adenomyosis, and 3 were excluded for not including a suitable control group. Ultimately, 25 studies were selected for inclusion in the meta-analysis (14–18, 20–23, 35–50) (Figure 1).

### 3.2 Characteristics and quality assessment results of the included studies

This investigation analyzed 25 cohort studies, comprising 20 retrospective and 5 prospective studies, which collectively included 24,113 women diagnosed with adenomyosis and 8,890,014 controls. The comparison group comprised women with normal uterine anatomy or no evidence of adenomyosis. While the majority of studies did not specify adenomyosis subtypes, some distinguished between diffuse and focal forms, with only one study exclusively addressing diffuse adenomyosis. The study population was categorized by ethnicity based on the countries where the studies were conducted: Caucasian (UK, Italy, France, Spain, The Netherlands, and Sweden), Asian (China, Japan, Korea, and India), and Mixed (USA, Israel, and Australia). Pregnant participants were categorized according to their method of conception, including natural conception, ART, or a combination of both. The study characteristics are detailed in Table 1. All included research was assessed as being of high quality, with detailed descriptions of study designs provided across the included literature (Supplementary Files 2).

**Table 1:** Characteristics of included studies.

Studies (year)	Study design	Country	Diagnosis of adenomyosis	Control population	Adenomyosis		Control		Adenomyosis type (diffuse or focal)	Mode of conception	Adjustments	Outcomes
					*N*	Age (years)	*N*	Age (years)				
Mavrelos et al. (2017) (44)	PCS	UK	Ultrasound scan	Women with normal uteri	72	36.0 (IQR 33.00–38.75)	303	34.0 (IQR 31.0–37.0)	Not specified	ART	Age, anti-Müllerian hormone, antral follicle count, and BMI	CPR and miscarriage
Matot et al. (2025) (17)	RCS	Israel	Three-dimensional transvaginal ultrasound	Patients without adenomyosis	59	34.0 (IQR 30.0–37.0)	62	30.0 (IQR 27.0–33.0)	Not specified	Natural conception	Maternal age, parity and aspirin usage	HDP
Costello et al. (2011) (38)	RCS	Australia	Pelvic ultrasound	Patients without adenomyosis	37	39.0 (range 27–42)	164	34.6 (range 18–42)	Diffuse and focal	ART	NR	CPR, LBR and IR
Ni et al. (2024) (23)	RCS	China	Ultrasound examination	Women without adenomyosis	321	≥ 18	10,507	All age groups	Not specified	ART and natural conception	Maternal age, parity, mode of conception, number of prior pregnancy losses < 14 weeks of gestation and number of prior pregnancy losses between 14 and 28 weeks of gestation	PTB, PP, and pre-eclampsia
Benaglia et al. (2014) (36)	PCS	Italy	Transvaginal ultrasound	Women without adenomyosis	49	35 ± 4	49	35 ± 4	Diffuse and focal	ART	BMI	CPR, LBR and IR
Liu et al. (2025) (22)	RCS	China	Ultrasound or MRI	Women without adenomyosis	413	35.11 ± 3.67	880	34.85 ± 3.45	Diffuse and focal	ART and natural conception	NR	PTB, CS, LBW and PP
Neal et al. (2020) (14)	PCS	USA	Three-dimensional ultrasound	Patients without adenomyosis	99	37.1 ± 5.2	549	35.9 ± 4.6	Not specified	ART	Age at transfer, BMI, previous failed transfer, endometrial preparation protocol and blastocyst quality	CPR, LBR and miscarriage
Bourdon et al. (2022) (37)	RCS	France	Pelvic magnetic resonance imaging	Endometriosis patients without adenomyosis	145	18–42	57	18–42	Diffuse and focal	ART	Women age, anti-Müllerian hormone level, length of infertility, and having a previous surgery for endometriosis	LBR
Mochimaru et al. (2015) (45)	RCS	Japan	MRI or ultrasonography	Women without adenomyosis	36	35.0 (range 27–43)	144	36.0 (range 25–43)	Diffuse and focal	ART and natural conception	NR	PTB, SGA, CS, pre-eclampsia and PPROM
Jung et al. (2024) (42)	RCS	Korea	ICD-10	Participants without adenomyosis	10,356	35.05 ± 4.03	1,210,178	32.94 ± 4.2	Not specified	ART and natural conception	Age, parity, hypertension before pregnancy, overt diabetes, pregnancy-associated hypertension, gestational diabetes, and myoma before pregnancy	PTB, SGA, CS, PP, PH and HDP
Yamaguchi et al. (2019) (49)	RCS	Japan	Assessing participants for a history of adenomyosis diagnosis	Participants without adenomyosis	311	35.0 ± 4.4	93,210	NR	Not specified	ART and natural conception	Maternal age, smoking status, method of conception, primiparity, fibroids, endometriosis and body mass index before pregnancy	PTB, SGA and LBW
Liang et al. (2022) (43)	RCS	China	Transvaginal ultrasonography	Women with normal uterus	1,146	34.82 ± 3.905	1,146	34.82 ± 3.905	Not specified	ART	Maternal age, number of embryos transferred, singleton and twin pregnancy	CPR, LBR, IR, miscarriage, PTB, PP, LBW, and pre-eclampsia
Shin et al. (2018) (16)	RCS	USA	Ultrasonographic examinations	Women without adenomyosis	72	34.1 ± 4.1	8,244	33.8 ± 3.6	Not specified	ART and natural conception	NR	PTB and LBW
Trinchant et al. (2025) (21)	RCS	Spain	Transvaginal ultrasound	Patients without adenomyosis	228	39.32 (95% CI 38.81–39.87)	228	38.90 (95% CI 38.48–39.36)	Diffuse and focal	ART	Age, BMI, smoking status, presence of endometriosis, presence of fibroids, previous deliveries and cesarean sections	CPR, LBR, IR, miscarriage, PTB, CS, and LBW
Yan et al. (2014) (50)	RCS	China	Transvaginal ultrasound scans	Patients without adenomyosis	77	34.18 ± 4.19	77	34.23 ± 4.17	Not specified	ART	Mean day 3 estrogen, total dosage of gonadotropin per cycle, duration of gonadotropin stimulation, and with or without endometriosis	CPR and miscarriage
Rees et al. (2023) (18)	RCS	The Netherlands	Histological diagnosis	Women without adenomyosis from the general population	7,925	29.15 ± 4.43	4,615,803	30.42 ± 4.87	Not specified	ART and natural conception	Maternal age, parity, ethnicity, year of registered birth, induction of labor, multiple gestation, and low socioeconomic status	Miscarriage, PTB, SGA, CS, PP, PH, HDP, and PPROM
Thalluri et al. (2012) (48)	RCS	Australia	Pelvic ultrasound	Women without adenomyosis	38	35 (IQR 32.7–37.3)	175	33 (IQR 30–36)	Not specified	ART	Age	CPR
Han et al. (2023) (40)	RCS	China	Ultrasound examination	Tubal infertility patients without adenomyosis	Diffuse: 428; Focal: 718	Diffuse: 35.50 ± 3.83; Focal: 34.42 ± 3.90	519	33.33 ± 3.90	Diffuse and focal	ART	Age, FSH level, body mass index, antral follicle count, anti-Müllerian hormone, the number of transferred embryos, uterine volume and endometrial thickness on transfer day	CPR, LBR and miscarriage
Scala et al. (2018) (15)	RCS	Italy	Ultrasonographic diagnosis	Endometriosis women without adenomyosis	Diffuse: 20; Focal: 38	Diffuse: 31 (IQR 27–33); Focal: 30 (IQR 26.5–33)	148	30.0 (IQR 27.0–33.0)	Diffuse and focal	ART and natural conception	BMI, pregnancy-associated plasma protein-A, and Second-trimester mean UtA-pulsatility index	SGA
Hou et al. (2020) (41)	RCS	China	Vaginal ultrasound	Patients with simple tubal factors treated with a long GnRH agonist protocol	127	31.8 (IQR 29–34)	3,471	31.6 (IQR 29–34)	Not specified	ART	Age, infertility duration, basal FSH, antral follicle count, and body mass index	CPR, LBR, IR and miscarriage
Alson et al. (2024) (35)	PCS	Sweden	Transvaginal ultrasonography	Women without adenomyosis	102	34.4 ± 3.8	935	31.7 ± 3.9	Diffuse and focal	ART	Age	CPR and LBR
Sharma et al. (2019) (46)	RCS	India	Two-dimensional transvaginal sonography	Women with tubal factor infertility	64	32.89 ± 2.98	466	33.02 ± 3.4	Diffuse	ART	NR	CPR, LBR and miscarriage
Cozzolino et al. (2024) (39)	PCS	Italy	Transvaginal sonography	Patients without adenomyosis	114	42.96 ± 3.34	114	43.02 ± 3.37	Diffuse and focal	ART	Age of patients, age of donors, BMI, sperm concentration, fresh or frozen sperm, HRT with agonist or antagonist, endometrial thickness, day of endometrial preparation, and indication for egg donation	LBR, IR and miscarriage
Stanekova et al. (2018) (47)	RCS	Australia	Pelvic ultrasound and/or MRI	Patients without adenomyosis	34	37.0 ± 4.0	137	35.9 ± 4.6	Diffuse and focal	ART	Age, BMI, and day 16 βhCG	Miscarriage
Hguig et al. (2025) (20)	RCS	USA	ICD-10-CM	Individuals without adenomyosis	1,084	All age groups	2,942,448	All age groups	Not specified	ART and natural conception	Age, ethnicity, hospital location/teaching status, insurance type, median household income quartile, comorbidities, and mode of delivery	PTB, pre-eclampsia, PH and PPROM

PCS, prospective cohort study; RCS, retrospective cohort study; IQR, interquartile range; ART, assisted reproductive technology; NR, not reported; MRI, magnetic resonance imaging; ICD-10-CM, International Classification of Diseases 10th Revision, Clinical Modification; CI, confidence interval; GnRH, gonadotrophin-releasing hormone; CPR, clinical pregnancy rate; LBR, live birth rate; IR, implantation rate; PTB, preterm birth; SGA, small for gestational age; PP, placenta previa; CS, cesarean section; LBW, low birth weight; PH, postpartum hemorrhage; HDP, hypertensive disorders of pregnancy; PPROM, preterm premature rupture of membranes.

### 3.3 Meta-analysis of primary outcomes

#### 3.3.1 Clinical pregnancy rate

**Figure 2:**
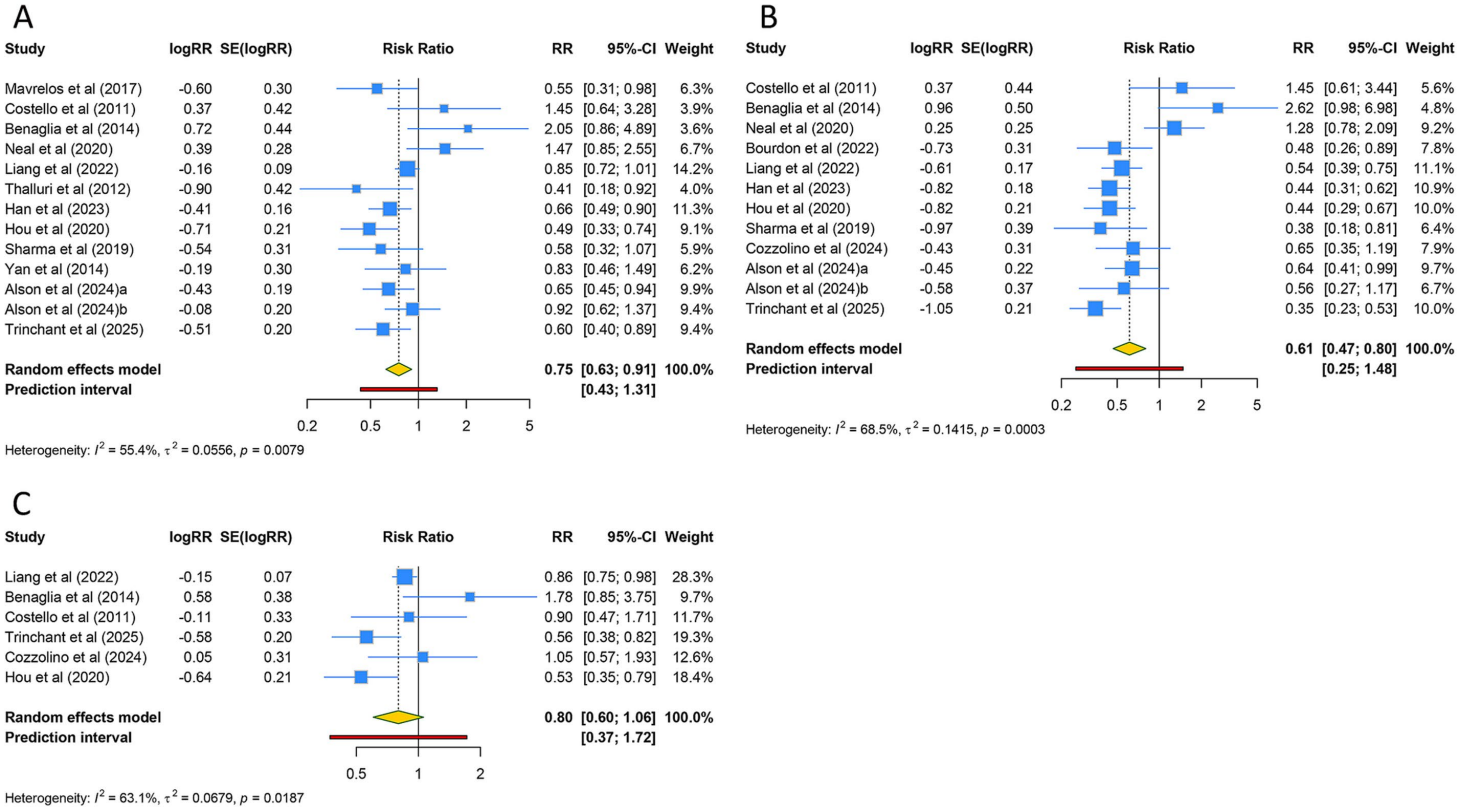
Forest plots of the association between adenomyosis and **(A)** clinical pregnancy rate, **(B)** live birth rate, and **(C)** implantation rate.

13 studies reported the clinical pregnancy rate. Due to relatively high heterogeneity among the included studies (I^2^ = 55.4%), a random-effects model was applied. The overall analysis revealed that women with adenomyosis had a significantly lower clinical pregnancy rate compared to the control population (RR [95% CI] = 0.753 [0.625–0.907], 95% PI: 0.433–1.311) (Table 2; Figure 2A). Subgroup analysis based on ethnicity indicated that this significant reduction in clinical pregnancy rate was observed only in Asian populations (RR [95% CI] = 0.750 [0.656–0.858], 95% PI: 0.395–1.224), whereas no statistically significant difference was found in Caucasian or mixed-ethnicity populations (all *p* > 0.05). Since the mode of conception in all 13 included studies was ART, subgroup analysis by mode of conception yielded results consistent with the overall analysis (Table 2; Supplementary Figure S1).

**Table 2:** Meta-analysis of the association between adenomyosis and primary outcomes.

Outcomes and subgroups	Number of studies	Meta-analysis				Heterogeneity	
		RR	95% CI	*p* value	95% PI	I^2^, Tau^2^	*p* value
Clinical pregnancy rate	13	0.753	0.625–0.907	0.003	0.433–1.311	55.4%, 0.056	0.008
Ethnicity							
Caucasian	5	0.755	0.549–1.040	0.085	0.319–1.790	55.4%, 0.070	0.062
Asian	5	0.750	0.656–0.858	<0.001	0.395–1.224	47.3%, 0.029	0.108
Mixed	3	0.983	0.446–2.165	0.965	0.045–21.377	72.2%, 0.350	0.027
Mode of conception							
ART	13	0.753	0.625–0.907	0.003	0.433–1.311	55.4%, 0.056	0.008
Live birth rate	12	0.613	0.469–0.801	<0.001	0.254–1.479	68.5%, 0.142	<0.001
Ethnicity							
Caucasian	6	0.616	0.405–0.938	0.024	0.184–2.068	67.1%, 0.176	0.010
Asian	4	0.471	0.386–0.575	<0.001	0.341–0.651	0%, 0	0.747
Mixed	2	1.320	0.861–2.021	0.203	0.083–20.941	0%, 0	0.806
Mode of conception							
ART	12	0.613	0.469–0.801	<0.001	0.254–1.479	68.5%, 0.142	<0.001
Implantation rate	6	0.800	0.604–1.059	0.119	0.372–1.717	63.1%, 0.068	0.019
Ethnicity							
Caucasian	3	0.958	0.488–1.883	0.902	0.066–13.948	76.4%, 0.269	0.014
Asian	2	0.700	0.437–1.121	0.138	0.005, 99.752	79.6%, 0.095	0.027
Mixed	1	0.900	0.473–1.712	0.748			
Mode of conception							
ART	6	0.800	0.604–1.059	0.119	0.372–1.717	63.1%, 0.068	0.019
Miscarriage	12	2.296	1.724–3.058	<0.001	0.975–5.406	70.0%, 0.130	<0.001
Ethnicity							
Caucasian	4	2.068	0.899–4.758	0.088	0.146–29.201	81.8%, 0.511	0.001
Asian	6	2.255	1.811–2.808	<0.001	1.383–3.970	21.5%, 0.024	0.272
Mixed	2	2.089	0.746–5.852	0.161	-	64.3%, 0.359	0.094
Mode of conception							
ART	11	2.339	1.930–2.834	<0.001	1.095–5.629	48.8%, 0.112	0.034
ART and natural conception	1	1.530	1.443–1.623	<0.001			
Preterm birth	10	2.006	1.390–2.894	<0.001	0.557–7.224	96.6%, 0.286	<0.001
Ethnicity							
Caucasian	2	1.736	0.278–10.846	0.555	-	87.6%, 1.554	0.005
Asian	6	2.133	1.738–2.617	<0.001	1.258–3.615	55.6%, 0.031	0.046
Mixed	2	2.020	0.866–4.710	0.104	-	82.3%, 0.316	0.017
Mode of conception							
ART	2	2.226	0.630–7.865	0.214	-	72.2%, 0.631	0.058
ART and natural conception	8	2.006	1.340–3.004	0.001	0.509–7.915	97.3%, 0.295	<0.001

ART, assisted reproductive technology.

#### 3.3.2 Live birth rate

A total of 12 studies assessed the live birth rate. Given the significant heterogeneity across the included studies (I^2^ = 68.5%), a random-effects model was utilized for the analysis. The findings demonstrated that women diagnosed with adenomyosis experienced a markedly lower live birth rate compared to the control group (RR [95% CI] = 0.613 [0.469–0.801], 95% PI: 0.254–1.479) (Table 2; Figure 2B). Ethnicity-based subgroup analyses revealed that this reduction remained statistically significant in both Caucasian and Asian populations (all *p* < 0.05), while no notable difference was identified in mixed-ethnicity groups (RR [95% CI] = 1.320 [0.861–2.021], 95% PI: 0.083–20.941). Notably, as all included studies were conducted within ART cohorts, stratification by conception method produced findings consistent with the overall analysis (Table 2; Supplementary Figure S2).

#### 3.3.3 Implantation rate

Six studies reported the implantation rate. Due to substantial heterogeneity among the included studies (I^2^ = 63.1%), a random-effects model was applied. The overall analysis showed no significant difference in implantation rate between women with adenomyosis and controls (RR [95% CI] = 0.800 [0.604–1.059], 95% PI: 0.372–1.717) (Table 2; Figure 2C). Subgroup analyses based on ethnicity and mode of conception also revealed no statistically significant results (all *p* > 0.05) (Table 2; Supplementary Figure S3).

#### 3.3.4 Miscarriage

**Figure 3:**
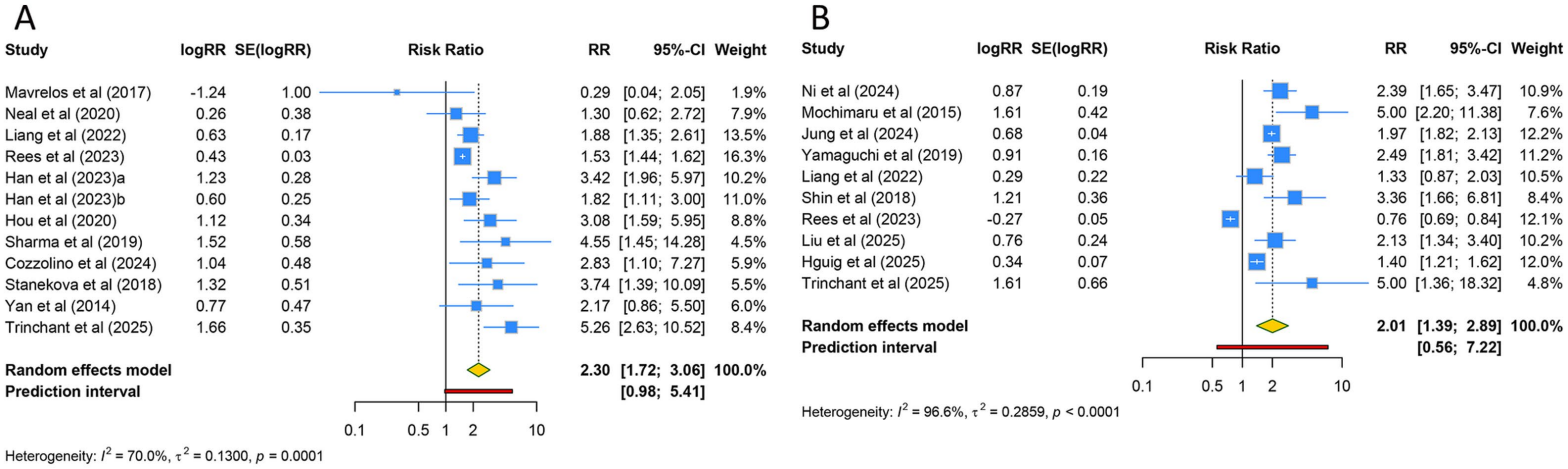
Forest plots of the association between adenomyosis and **(A)** miscarriage and **(B)** preterm birth.

12 studies assessed miscarriage in adenomyosis women. Due to substantial heterogeneity among the included studies (I^2^ = 70.0%), a random-effects model was applied. The overall analysis revealed that women with adenomyosis had a significantly increased risk of miscarriage compared to controls (RR [95% CI] = 2.296 [1.724–3.058], 95% PI: 0.975–5.406) (Table 2; Figure 3A). Subgroup analysis based on ethnicity indicated that this significant increase in miscarriage was observed only in Asian populations (RR [95% CI] = 2.255 [1.811–2.808], 95% PI: 1.383–3.970), whereas no statistically significant difference was found in Caucasian or mixed-ethnicity populations (all *p* > 0.05). Subgroup analysis based on the mode of conception indicated that the miscarriage rate was significantly higher among women with adenomyosis in both the ART subgroup and the ART and natural conception subgroup (all *p* < 0.05) (Table 2; Supplementary Figure S4).

#### 3.3.5 Preterm birth

Preterm birth outcome in women with adenomyosis were examined in 10 studies. With substantial heterogeneity necessitating the use of a random-effects model (I^2^ = 96.6%). The pooled analysis indicated that adenomyosis was associated with a markedly higher risk of preterm birth compared to the control group (RR [95% CI] = 2.006 [1.390–2.894], 95% PI: 0.557–7.224) (Table 2; Figure 3B). Ethnicity-based subgroup analyses revealed that this heightened risk was confined to Asian populations (RR [95% CI] = 2.133 [1.738–2.617], 95% PI: 1.258–3.615), while no statistically significant differences were observed in Caucasian or mixed-ethnicity cohorts (all *p* > 0.05). When analyzed by conception method, the increased risk of preterm birth was significant only in the ART and natural conception subgroup (RR [95% CI] = 2.006 [1.340–3.004], 95% PI: 0.509–7.915) (Table 2; Supplementary Figure S5).

### 3.4 Meta-analysis of secondary outcomes

#### 3.4.1 SGA and placenta previa

**Figure 4:**
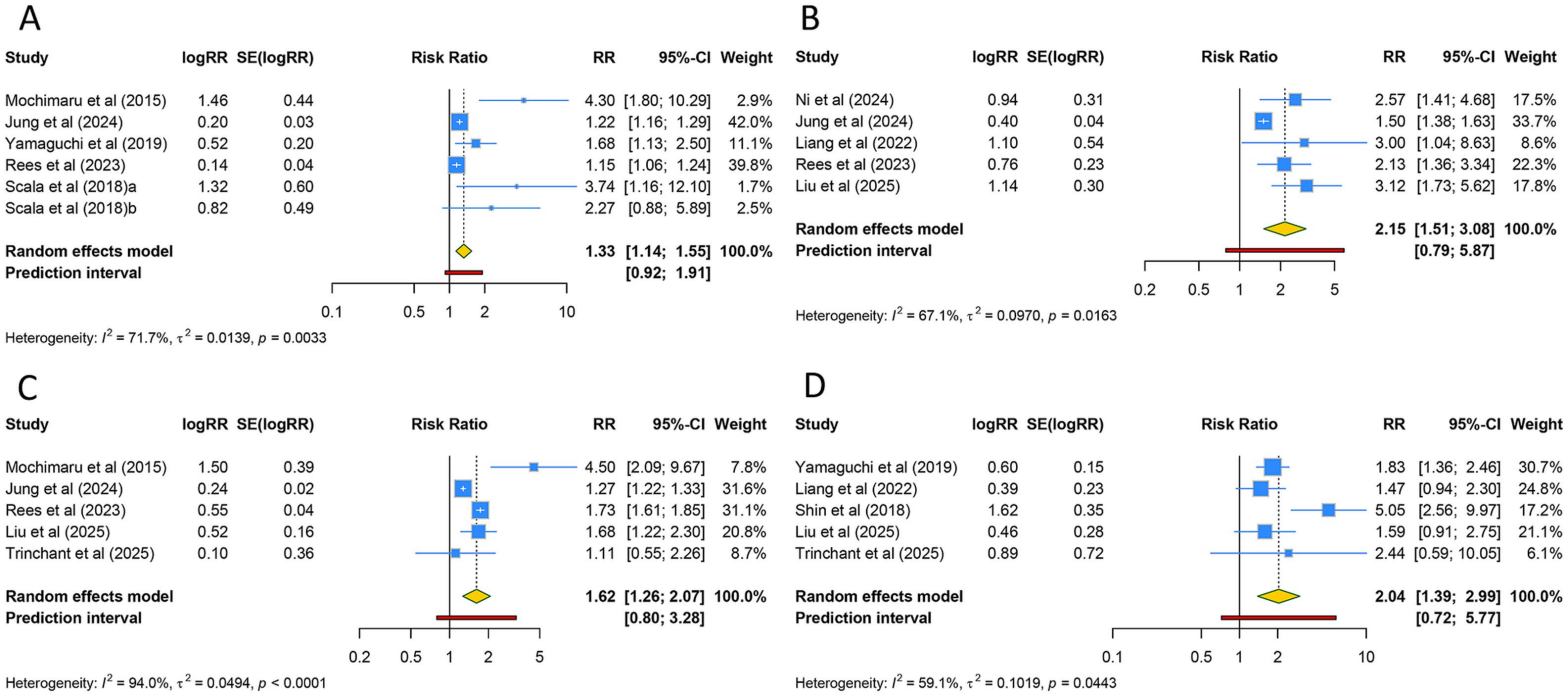
Forest plots of the association between adenomyosis and **(A)** small for gestational age, **(B)** placenta previa, **(C)** cesarean section, and **(D)** low birth weight.

Six studies reported on SGA. The overall analysis using a random-effects model (I^2^ = 71.7%) revealed that the incidence of SGA was significantly higher in women with adenomyosis compared with controls (RR [95% CI] = 1.325 [1.136–1.546], 95% PI: 0.921–1.908) (Table 3; Figure 4A). This significant increase was observed only in Asian populations (RR [95% CI] = 1.742 [1.052–2.886], 95% PI: 0.241–12.623), while no significant difference was found in Caucasian populations (*p* > 0.05). Since the mode of conception in all six studies was ART and natural conception, subgroup analysis based on the mode of conception yielded results consistent with the overall analysis (Table 3; Supplementary Figure S6).

**Table 3:** Meta-analysis of the association between adenomyosis and secondary outcomes.

Outcomes and subgroups	Number of studies	Meta-analysis				Heterogeneity	
		RR	95% CI	*p* value	95% PI	I^2^, Tau^2^	*p* value
SGA	6	1.325	1.136–1.546	<0.001	0.921–1.908	71.7%, 0.014	0.003
Ethnicity							
Caucasian	3	1.797	0.865–3.729	0.116	0.114–28.375	65.5%, 0.273	0.055
Asian	3	1.742	1.052–2.886	0.031	0.241–12.623	80.7%, 0.146	0.006
Mode of conception							
ART and natural conception	6	1.325	1.136–1.546	<0.001	0.921–1.908	71.7%, 0.014	0.003
Placenta previa	5	2.154	1.506–3.081	<0.001	0.791–5.870	67.1%, 0.097	0.016
Ethnicity							
Caucasian	1	2.130	1.359–3.338	0.001			
Asian	4	2.232	1.393–3.576	0.001	0.526–9.461	70.7%, 0.148	0.017
Mode of conception							
ART	1	2.996	1.040–8.631	0.042			
ART and natural conception	4	2.091	1.434–3.048	<0.001	0.647–6.756	71.9%, 0.099	0.014
Cesarean section	5	1.617	1.264–2.068	<0.001	0.796–3.285	94.0%, 0.049	<0.001
Ethnicity							
Caucasian	2	1.723	1.608–1.846	<0.001	0.071–36.390	32.9%, 0.032	0.222
Asian	3	1.811	1.139–2.878	0.012	0.287–11.439	84.8%, 0.128	0.001
Mode of conception							
ART	1	1.110	0.546–2.255	0.773			
ART and natural conception	4	1.678	1.295–2.176	<0.001	0.732–3.848	95.4%, 0.050	<0.001
LBW	5	2.039	1.390–2.991	<0.001	0.721–5.766	59.1%, 0.102	0.044
Ethnicity							
Caucasian	1	2.440	0.593–10.045	0.217			
Asian	3	1.692	1.352–2.118	<0.001	1.034–2.770	0%, 0	0.707
Mixed	1	5.050	2.559–9.966	<0.001			
Mode of conception							
ART	2	1.542	1.007–2.363	0.047	0.097–24.501	0%, 0	0.505
ART and natural conception	3	2.317	1.299–4.134	0.004	0.237–22.702	75.5%, 0.194	0.017
Pre-eclampsia	4	1.296	1.078–1.558	0.006	0.719–2.755	20.6%, 0.023	0.287
Ethnicity							
Asian	3	1.868	1.200–2.907	0.006	0.707–4.935	0%, 0	0.739
Mixed	1	1.200	0.980–1.470	0.078			
Mode of conception							
ART	1	2.287	1.030–5.079	0.042			
ART and natural conception	3	1.255	1.038–1.517	0.019	0.828–1.902	0%, 0	0.423
Postpartum hemorrhage	3	1.507	1.053–2.156	0.025	0.323–7.023	96.6%, 0.095	<0.001
Ethnicity							
Caucasian	1	1.230	1.098–1.378	<0.001			
Asian	1	1.096	1.035–1.161	0.002			
Mixed	1	2.700	2.154–3.385	<0.001			
Mode of conception							
ART and natural conception	3	1.507	1.053–2.156	0.025	0.323–7.023	96.6%, 0.095	<0.001
HDP	3	1.288	1.053–1.576	0.014	0.610–2.718	83.5%, 0.020	0.002
Ethnicity							
Caucasian	1	1.370	1.251–1.501	<0.001			
Asian	1	1.149	1.059–1.246	0.001			
Mixed	1	5.910	1.322–26.430	0.020			
Mode of conception							
Natural conception	1	5.910	1.322–26.430	0.020			
ART and natural conception	2	1.253	1.055–1.489	0.010	0.196–7.993	87.4%, 0.014	0.005
PPROM	3	1.459	1.098–1.940	0.009	0.527–4.045	65.4%, 0.035	0.055
Ethnicity							
Caucasian	1	1.410	1.158–1.717	0.001			
Asian	1	5.500	1.705–17.747	0.004			
Mixed	1	1.300	1.078–1.568	0.006			
Mode of conception							
ART and natural conception	3	1.459	1.098–1.940	0.009	0.527–4.045	65.4%, 0.035	0.055

SGA, small for gestational age; ART, assisted reproductive technology; LBW, low birth weight; HDP, hypertensive disorders of pregnancy; PPROM, preterm premature rupture of membranes.

Five studies evaluated the risk of placenta previa in women with adenomyosis. The overall analysis using a random-effects model (I^2^ = 67.1%) demonstrated that women with adenomyosis had a significantly increased risk of placenta previa compared with controls (RR [95% CI] = 2.154 [1.506–3.081], 95% PI: 0.791–5.870) (Table 3; Figure 4B). Subgroup analyses based on ethnicity and mode of conception further supported the significant increase in the risk of placenta previa (all *p* < 0.05) (Table 3; Supplementary Figure S7).

#### 3.4.2 Cesarean section and LBW

A total of five investigations examined the likelihood of cesarean section among individuals diagnosed with adenomyosis. Utilizing a random-effects model for the meta-analysis (I^2^ = 94.0%), findings indicated that the probability of undergoing cesarean section was markedly elevated in adenomyosis patients compared to those without the condition (RR [95% CI] = 1.617 [1.264–2.068], 95% PI: 0.796–3.285) (Table 3; Figure 4C). This heightened risk was evident across both Asian and Caucasian groups (all *p* < 0.05). Further stratification by conception method indicated that this heightened risk was confined to pregnancies achieved via ART and natural conception (RR [95% CI] = 1.678 [1.295–2.176], 95% PI: 0.732–3.848) (Table 3; Supplementary Figure S8).

The relationship between adenomyosis and the incidence of LBW was evaluated in five studies. The aggregated results, derived from a random-effects model (I^2^ = 59.1%), demonstrated a markedly higher prevalence of LBW among women with adenomyosis relative to the control group (RR [95% CI] = 2.039 [1.390–2.991], 95% PI: 0.721–5.766) (Table 3; Figure 4D). This association was particularly pronounced in Asian cohorts and populations of mixed ethnicity (all *p* < 0.05). Subgroup analyses based on conception modality revealed that the elevated risk of LBW was significant in pregnancies conceived through ART alone, as well as in those conceived via both ART and natural methods (all *p* < 0.05) (Table 3; Supplementary Figure S9).

#### 3.4.3 Pre-eclampsia and postpartum hemorrhage

**Figure 5:**
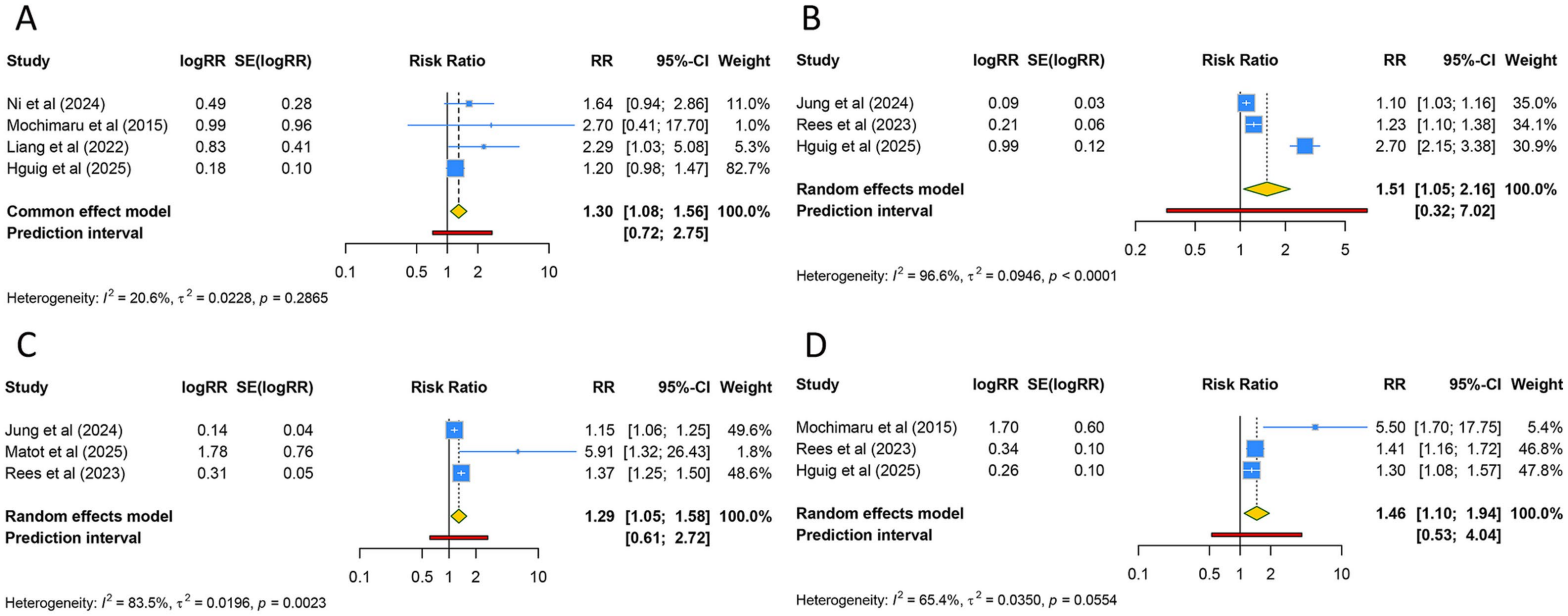
Forest plots of the association between adenomyosis and **(A)** pre-eclampsia, **(B)** postpartum hemorrhage, **(C)** hypertensive disorders of pregnancy, and **(D)** preterm premature rupture of membranes.

Four studies reported on the risk of pre-eclampsia in women with adenomyosis. The pooled analysis using a fixed-effects model (I^2^ = 20.6%) revealed that the risk of pre-eclampsia was significantly higher in women with adenomyosis compared with controls (RR [95% CI] = 1.296 [1.078–1.558], 95% PI: 0.719–2.755) (Table 3; Figure 5A). This significant increase was observed exclusively in Asian populations (*p* < 0.05). Subgroup analysis based on the mode of conception indicated that the risk of pre-eclampsia was significantly elevated in both the ART subgroup and the ART and natural conception subgroup (all *p* < 0.05) (Table 3; Supplementary Figure S10).

Three studies evaluated the incidence of postpartum hemorrhage. The overall analysis using a random-effects model (I^2^ = 96.6%) demonstrated that the incidence of postpartum hemorrhage was significantly higher in women with adenomyosis compared with controls (RR [95% CI] = 1.507 [1.053–2.156], 95% PI: 0.323–7.023) (Table 3; Figure 5B). This significant increase was observed across Caucasian, Asian, and mixed-ethnicity populations, although only one study was included in each ethnic subgroup. Since all three included studies were categorized under the ART and natural conception group, subgroup analysis based on the mode of conception yielded results identical to the overall analysis (Table 3; Supplementary Figure S11).

#### 3.4.4 HDP and PPROM

Three studies reported on the risk of HDP in women with adenomyosis. The pooled analysis using a random-effects model (I^2^ = 83.5%) revealed that the risk of HDP was significantly higher in women with adenomyosis compared with controls (RR [95% CI] = 1.288 [1.053–1.576], 95% PI: 0.610–2.718) (Table 3; Figure 5C). This elevated risk was evident across Caucasian, Asian, and mixed-ethnicity populations (all *p* < 0.05), although each subgroup analysis was based on a single study. Stratified assessment by conception method revealed that the heightened risk was significant in pregnancies conceived naturally as well as in those involving both ART and natural conception (all *p* < 0.05) (Table 3; Supplementary Figure S12).

The incidence of PPROM in women with adenomyosis was investigated in three studies. Pooled estimates from the random-effects model (I^2^ = 65.4%) demonstrated a significantly higher occurrence of PPROM in women with adenomyosis compared to controls (RR [95% CI] = 1.459 [1.098–1.940], 95% PI: 0.527–4.045) (Table 3; Figure 5D). This increase was also observed across Caucasian, Asian, and mixed-ethnicity groups (all *p* < 0.05), despite each subgroup analysis being limited to one study. Notably, as all three studies were classified under the ART and natural conception group, the results of the subgroup analysis aligned entirely with those of the overall evaluation (Table 3; Supplementary Figure S13).

### 3.5 Sensitivity analysis and publication bias

Sensitivity and publication bias analyses were conducted for pooled outcomes derived from datasets comprising ten or more studies. The leave-one-out sensitivity analysis revealed that removing any single study did not significantly alter the overall conclusions for clinical pregnancy rate, live birth rate, miscarriage, or preterm birth, highlighting the robustness and consistency of these results (Supplementary Figure S14). While Begg’s and Egger’s tests suggested the possibility of publication bias in the miscarriage outcome (Begg’s test: *p* = 0.732; Egger’s test: *p* = 0.022), subsequent adjustment using the trim-and-fill method revealed no significant alterations in either the direction or statistical significance of the results, indicating that the conclusions remained largely unaffected by the potential bias. No evidence of publication bias was detected for the other outcomes. Funnel plots corresponding to these analyses are provided in Supplementary Figure S15.

## 4 Discussion

The effects of adenomyosis on pregnancy complications have attracted considerable attention in recent years. Previous research examining its role in perinatal outcomes has produced inconsistent findings (16, 45, 51–55). This investigation consolidated data from various cohort studies to assess its impact on a broad spectrum of maternal, fetal, and obstetric outcomes. In terms of reproductive health, the meta-analysis revealed that women diagnosed with adenomyosis experience notably reduced rates of clinical pregnancy and live births compared to those without the condition. Additionally, they face elevated risks of miscarriage and preterm birth. Moreover, adenomyosis is associated with an increased risk of SGA, placenta previa, cesarean section, LBW, pre-eclampsia, postpartum hemorrhage, HDP, and PPROM.

A prior meta-analysis conducted by Younes et al. identified a significant association between adenomyosis and both diminished pregnancy rate and reduced live birth rate (56), a conclusion that aligns with the observations of the present study. Traditionally considered a uterine disorder predominantly affecting multiparous women, growing evidence has linked adenomyosis to infertility and compromised reproductive outcomes (56). This condition alters the eutopic endometrium, disrupts the uterine environment, and impairs contractility, all of which diminish the uterus’s ability to support embryo implantation and subsequent development, leading to infertility and suboptimal implantation success (57, 58). The adverse effects on embryo implantation mechanisms further contribute to reduced live birth rates (59), as adenomyosis interferes with key biological processes critical for conception and pregnancy progression. Additionally, heightened uterine peristalsis associated with adenomyosis can displace embryos during implantation (60), complicating early pregnancy. Patients with adenomyosis also exhibit elevated oxidative stress within the endometrium, which may result in cellular damage, impairing endometrial quality and functionality (61). These changes negatively impact embryo implantation and development, increasing the risk of implantation failure and complications in later pregnancy stages. However, our analysis did not identify a significant difference in implantation rate between patients with adenomyosis and those without. Given the limited number of studies included in the analysis, this finding warrants further validation and exploration.

Numerous studies have indicated that women with adenomyosis face a higher likelihood of experiencing miscarriage and preterm birth (12, 45, 62, 63). Research consistently highlights an elevated risk of pregnancy loss in patients with this condition (47, 64). Recent independent findings further confirm that miscarriage rates are significantly higher in women with adenomyosis compared to those without (51), reinforcing earlier reports of diminished clinical pregnancy (65) and live birth rates in individuals exhibiting three distinct adenomyotic features (64). The association between adenomyosis and preterm birth is thought to arise from its underlying pathophysiology, which includes chronic uterine inflammation (66), elevated prostaglandin levels (53), and increased intrauterine pressure (67, 68). Khan et al. (69) demonstrated that the endometrial tissue of women with adenomyosis exhibits heightened inflammatory activity compared to healthy controls. Additional research has revealed that large adenomyotic lesions are associated with a greater risk of cervical incompetence, likely due to excessive intrauterine pressure and inflammatory processes (55). Moreover, elevated levels of inflammatory mediators, including prostaglandins and cytokines associated with preterm birth, have been observed in patients with adenomyosis (70). These inflammatory factors are thought to promote both localized and systemic inflammation, resulting in myometrial vasoconstriction, cervical remodeling, and irritation of the uterine and fetal membranes (53, 71). This cascade of events, coupled with impaired collagen synthesis, increases the risk of PPROM (53) and subsequent pregnancy complications.

Adenomyosis has been implicated in raising the likelihood of adverse pregnancy and neonatal outcomes, such as pre-eclampsia and SGA infants (13), through various pathological mechanisms. Key disruptions include altered uterine junctional zone (JZ) integrity, which impairs uterine peristalsis during the luteal phase (18, 72, 73), and heightened intrauterine oxidative stress, which may drive maternal endothelial dysfunction and abnormal placentation (74, 75). Additionally, an inflammatory intrauterine environment has been implicated in impaired myometrial decidualization, potentially leading to defective trophoblastic invasion of the JZ during pregnancy (76). Emerging evidence suggests that the elevated prevalence of HDP in women with adenomyosis could be attributed to suboptimal spiral artery remodeling within the JZ (77–79). Furthermore, the elevated risks of preterm birth and SGA associated with adenomyosis may also contribute to a greater prevalence of LBW infants. Studies have also highlighted a connection between fetal growth restriction (FGR) and adenomyosis, potentially due to severe placental abnormalities and reduced placental perfusion observed in affected pregnancies (77). This impaired placental function could partially explain the connection between FGR and LBW. Adenomyosis also disrupts the structural integrity of the uterine JZ, adversely affecting uterine peristalsis during the luteal phase, which is essential for proper implantation (80). This abnormal uterine contractility has been associated with complications such as placenta previa and postpartum hemorrhage (18, 81). Additionally, our research identified a marked rise in cesarean section rate among pregnancies affected by adenomyosis. This outcome may be attributed to uterine enlargement caused by the condition, which reduces the available intrauterine space. Such structural changes are likely to increase the likelihood of abnormal fetal positioning, ultimately necessitating surgical intervention (45).

ART pregnancies are inherently associated with an elevated risk of adverse outcomes, including pre-eclampsia and LBW (82, 83), making it challenging to discern whether the adverse outcomes observed in cases of adenomyosis are attributable to the condition itself or are a consequence of ART conception. Therefore, the current study stratified all outcomes based on the mode of conception. Notably, the reduced clinical pregnancy and live birth rates, alongside heightened risks of miscarriage, placenta previa, LBW, and pre-eclampsia, remained evident in patients with adenomyosis after ART. However, as only one included study reported pregnancy outcomes following natural conception, our subgroup analysis was insufficient to fully compare maternal and perinatal outcomes between patients with adenomyosis undergoing ART and those conceiving naturally. Consequently, it remains unclear whether the above pregnancy complications are attributable to adenomyosis itself or to ART. Nonetheless, research indicates that different ART protocols may indeed influence pregnancy and neonatal outcomes in the general population (84, 85), and it is plausible that these effects could be modified by the presence of adenomyosis. This highlights the need for additional subgroup analyses based on ART treatments types (e.g., *in vitro* fertilization [IVF] vs. intracytoplasmic sperm injection [ICSI]) in future studies to further elucidate these associations. In addition, unlike the lack of statistical significance observed in Caucasian populations, adenomyosis remained significantly associated with a lower clinical pregnancy rate and increased risks of miscarriage, preterm birth, SGA, and LBW in Asian populations. The stronger associations observed in Asian populations should be interpreted with caution, as they may reflect differences in healthcare systems, diagnostic practices, and reproductive technologies rather than true biological or causal effects. These potential confounders highlight the need for careful consideration of contextual factors and underscore the importance of standardized methodologies in future multiethnic studies. Moreover, the high heterogeneity (I^2^ > 90%) observed in the associations between adenomyosis and preterm birth, cesarean section, and postpartum hemorrhage likely reflects variations in diagnostic criteria for adenomyosis, including differences in imaging modalities and disease severity. Additionally, heterogeneity may arise from differences in study design, population demographics, and adjustments for confounding factors. These methodological and clinical discrepancies limit the generalizability of pooled estimates. Thus, while the findings suggest potential associations, cautious interpretation is warranted, emphasizing the need for standardized diagnostic protocols and more robustly controlled studies to refine these estimates.

This study is subject to several limitations. First, the diagnostic methods for adenomyosis, adenomyosis subtypes, mode of conception, and adjustments for confounding factors varied across studies, which largely contributed to the substantial heterogeneity observed in most outcomes. Second, some of the data used in the analysis were not derived from multivariable logistic regression models that accounted for confounding factors, potentially leading to residual bias or instability due to unmeasured variables. However, sensitivity analyses revealed that the results of this study remained consistent and reliable. Third, for outcomes with relatively high incidence rates, such as clinical pregnancy, live birth, implantation, and cesarean section, treating ORs as RRs in the pooled analysis may overestimate the strength of the association between adenomyosis and these outcomes. For these high-incidence outcomes, more cohort studies reporting RRs are needed to further validate our findings. Fourth, due to the lack of clear differentiation between adenomyosis subtypes and severity in most included studies, subgroup analyses stratifying by these factors could not be conducted to evaluate their distinct effects on reproductive and pregnancy outcomes. Additionally, certain subgroup analyses, particularly those addressing secondary outcomes, were based on a limited number of studies. As a result, the statistical strength of these evaluations is constrained, necessitating cautious interpretation of the results. This underscores the importance of conducting more cohort studies with detailed and comprehensive data to validate these findings. Fifth, adenomyosis and endometriosis frequently occur together, as they share overlapping pathophysiological features (86). Notably, endometriosis has been linked to negative obstetric outcomes, such as higher risks of preterm birth, pre-eclampsia, and placenta previa (87). In this meta-analysis, however, the studies analyzed did not clearly separate cases of adenomyosis from those involving concurrent endometriosis, which may have affected the reported associations. Future studies should systematically differentiate between these two conditions to provide a more precise understanding of their respective impacts on maternal and neonatal outcomes.

## 5 Conclusion

In conclusion, our findings suggest that women with adenomyosis have lower clinical pregnancy and live birth rates compared with those without adenomyosis and are at higher risk of adverse maternal and neonatal outcomes, including miscarriage, preterm birth, SGA, placenta previa, cesarean section, LBW, pre-eclampsia, postpartum hemorrhage, HDP, and PPROM. However, these associations should be interpreted cautiously due to the observational nature of the included studies and the substantial heterogeneity observed. Notably, the negative impact on reproductive outcomes, including reduced clinical pregnancy and live birth rates and elevated risks of miscarriage, placenta previa, LBW, and pre-eclampsia, appears to persist in patients undergoing ART. Further research is warranted to explore the influence of adenomyosis on pregnancy outcomes, with attention to adenomyosis subtypes, conception methods, and ethnic variations.

## Data Availability

The original contributions presented in the study are included in the article/Supplementary material, further inquiries can be directed to the corresponding author.
