# Supplementary material for: Association between adenomyosis and maternal and neonatal outcomes: a systematic review and meta-analysis
Source: Front Med (Lausanne). 2026 Mar 31;13:1772838. doi: 10.3389/fmed.2026.1772838 (PMC13076185; doi:10.3389/fmed.2026.1772838)
Supplement: Supplementary file 1 [file Table_1.docx]

| **TABLE S1** The Newcastle-Ottawa quality assessment scale for the included cohort studies. | | | | | | | | | | | | |
| --- | --- | --- | --- | --- | --- | --- | --- | --- | --- | --- | --- | --- |
| Study | Selection | | | |  | Comparability | |  | Assessment of outcome | | | Total score |
|  | Representativeness of exposure arm(s) | Selection of the comparative arm(s) | Origin of exposure source | Demonstration that outcome of interest was not present at start of study |  | Studies controlling the most important factors | Studies controlling the other main factors |  | Assessment of outcome with independency | Adequacy of follow-up length | Lost to follow-up acceptable |  |
| Mavrelos et al (2017) | 1 | 1 | 1 | 1 |  | 1 | 0 |  | 1 | 1 | 1 | 8 |
| Matot et al (2025) | 1 | 1 | 1 | 1 |  | 1 | 0 |  | 1 | 1 | 1 | 8 |
| Costello et al (2011) | 1 | 1 | 1 | 1 |  | 1 | 0 |  | 1 | 1 | 1 | 8 |
| Ni et al (2024) | 1 | 1 | 1 | 1 |  | 0 | 0 |  | 1 | 1 | 1 | 7 |
| Benaglia et al (2014) | 1 | 1 | 1 | 1 |  | 1 | 1 |  | 1 | 1 | 0 | 8 |
| Liu et al (2025) | 1 | 1 | 1 | 1 |  | 1 | 0 |  | 1 | 1 | 1 | 8 |
| Neal et al (2020) | 1 | 1 | 1 | 1 |  | 1 | 0 |  | 1 | 1 | 1 | 8 |
| Bourdon et al (2022) | 1 | 1 | 1 | 1 |  | 0 | 0 |  | 1 | 1 | 1 | 7 |
| Mochimaru et al (2015) | 1 | 1 | 1 | 1 |  | 1 | 0 |  | 1 | 1 | 1 | 8 |
| Jung et al (2024) | 1 | 1 | 1 | 1 |  | 1 | 0 |  | 1 | 1 | 1 | 8 |
| Yamaguchi et al (2019) | 1 | 1 | 1 | 1 |  | 1 | 0 |  | 1 | 1 | 1 | 8 |
| Liang et al (2022) | 1 | 1 | 1 | 1 |  | 1 | 0 |  | 1 | 1 | 1 | 8 |
| Shin et al (2018) | 1 | 1 | 1 | 1 |  | 1 | 0 |  | 1 | 1 | 1 | 8 |
| Trinchant et al (2025) | 1 | 1 | 1 | 1 |  | 1 | 0 |  | 1 | 1 | 1 | 8 |
| Yan et al (2014) | 1 | 1 | 1 | 1 |  | 1 | 1 |  | 1 | 1 | 1 | 9 |
| Rees et al (2023) | 1 | 1 | 1 | 1 |  | 1 | 0 |  | 1 | 1 | 1 | 8 |
| Thalluri et al (2012) | 1 | 1 | 1 | 1 |  | 1 | 0 |  | 1 | 1 | 1 | 8 |
| Han et al (2023) | 1 | 1 | 1 | 1 |  | 0 | 0 |  | 1 | 1 | 1 | 7 |
| Scala et al (2018) | 1 | 1 | 1 | 1 |  | 1 | 0 |  | 1 | 1 | 1 | 8 |
| Hou et al (2020) | 1 | 1 | 1 | 1 |  | 1 | 0 |  | 1 | 1 | 1 | 8 |
| Alson et al (2024) | 1 | 1 | 1 | 1 |  | 1 | 0 |  | 1 | 1 | 1 | 8 |
| Sharma et al (2019) | 1 | 1 | 1 | 1 |  | 1 | 0 |  | 1 | 1 | 1 | 8 |
| Cozzolino et al (2024) | 1 | 1 | 1 | 1 |  | 1 | 1 |  | 1 | 1 | 1 | 9 |
| Stanekova et al (2018) | 1 | 1 | 1 | 1 |  | 1 | 0 |  | 1 | 1 | 1 | 8 |
| Hguig et al (2025) | 1 | 1 | 1 | 1 |  | 0 | 0 |  | 1 | 1 | 1 | 7 |
