# Supplementary material for: Association between adenomyosis and maternal and neonatal outcomes: a systematic review and meta-analysis
Source: Front Med (Lausanne). 2026 Mar 31;13:1772838. doi: 10.3389/fmed.2026.1772838 (PMC13076185; doi:10.3389/fmed.2026.1772838)
Supplement: Supplementary file 2 [file Data_Sheet_1.docx]

**PubMed: 736**

**#1** (adenomyosis) OR (adenomyoses)

**#2** (pregnancy) OR (fertility) OR (neonatal outcomes) OR (obstetric outcomes) OR (reproductive outcomes) OR (spontaneous abortion) OR (miscarriage) OR (clinical pregnancy rate) OR (livebirth rate) OR (preterm birth) OR (preterm labor) OR (preterm delivery) OR (premature labor) OR (premature delivery) OR (premature birth) OR (miscarriage) OR (stillbirth) OR (cesarean section) OR (c-section) OR (caesarean) OR (low birth weight) OR (small for gestational age) OR (placenta previa) OR (placenta praevia) OR (pre-eclampsia) OR (preeclampsia) OR (hypertensive disorders in pregnancy) OR (gestational hypertension) OR (pregnancy induced hypertension) OR (postpartum hemorrhage) OR (spontaneous hemoperitoneum) OR (antepartum hemorrhage) OR (abruptio placentae) OR (placental abruption) OR (growth restriction) OR (fertilisation rate) OR (fertilization rate) OR (intestinal perforation) OR (bowel perforation) OR (cholestasis) OR (mid-trimester loss) OR (implantation rate) OR (ovarian response) OR (cycle cancellation)

**#3** (cohort) OR (prospective) OR (retrospective) OR (follow-up) OR (longitudinal)

**#4** #1 AND #2 AND #3

**Embase: 844**

**#1** 'adenomyosis'/exp OR adenomyoses

**#2** 'pregnancy'/exp OR 'fertility'/exp OR 'neonatal outcomes' OR 'obstetric outcomes' OR 'reproductive outcomes' OR 'spontaneous abortion'/exp OR 'clinical pregnancy rate'/exp OR 'livebirth rate' OR 'preterm birth'/exp OR 'preterm labor'/exp OR 'preterm delivery'/exp OR 'premature labor'/exp OR 'premature delivery'/exp OR 'premature birth'/exp OR 'miscarriage'/exp OR 'stillbirth'/exp OR 'cesarean section'/exp OR 'c section' OR caesarean OR 'low birth weight'/exp OR 'small for gestational age'/exp OR 'placenta previa'/exp OR 'placenta praevia'/exp OR 'pre eclampsia'/exp OR 'preeclampsia'/exp OR 'hypertensive disorders in pregnancy' OR 'gestational hypertension'/exp OR 'pregnancy induced hypertension'/exp OR 'postpartum hemorrhage'/exp OR 'spontaneous hemoperitoneum'/exp OR 'antepartum hemorrhage'/exp OR 'abruptio placentae'/exp OR 'placental abruption'/exp OR 'growth restriction' OR 'fertilisation rate' OR 'fertilization rate'/exp OR 'intestinal perforation'/exp OR 'bowel perforation'/exp OR 'cholestasis'/exp OR 'mid-trimester loss' OR 'implantation rate'/exp OR 'ovarian response'/exp OR 'cycle cancellation'

**#3** 'cohort'/exp OR prospective OR retrospective OR 'follow up'/exp OR 'longitudinal'/exp

**#4** #1 AND #2 AND #3

**Web of Science: 933**

**#1** TS=((adenomyosis) OR (adenomyoses))

**#2** TS=((pregnancy) OR (fertility) OR (neonatal outcomes) OR (obstetric outcomes) OR (reproductive outcomes) OR (spontaneous abortion) OR (miscarriage) OR (clinical pregnancy rate) OR (livebirth rate) OR (preterm birth) OR (preterm labor) OR (preterm delivery) OR (premature labor) OR (premature delivery) OR (premature birth) OR (miscarriage) OR (stillbirth) OR (cesarean section) OR (c-section) OR (caesarean) OR (low birth weight) OR (small for gestational age) OR (placenta previa) OR (placenta praevia) OR (pre-eclampsia) OR (preeclampsia) OR (hypertensive disorders in pregnancy) OR (gestational hypertension) OR (pregnancy induced hypertension) OR (postpartum hemorrhage) OR (spontaneous hemoperitoneum) OR (antepartum hemorrhage) OR (abruptio placentae) OR (placental abruption) OR (growth restriction) OR (fertilisation rate) OR (fertilization rate) OR (intestinal perforation) OR (bowel perforation) OR (cholestasis) OR (mid-trimester loss) OR (implantation rate) OR (ovarian response) OR (cycle cancellation))

**#3** TS=((cohort) OR (prospective) OR (retrospective) OR (follow-up) OR (longitudinal))

**#4** #1 AND #2 AND #3

**The Cochrane Library: 117**

**#1** All Text=((adenomyosis) OR (adenomyoses))

**#2** All Text=((pregnancy) OR (fertility) OR (neonatal outcomes) OR (obstetric outcomes) OR (reproductive outcomes) OR (spontaneous abortion) OR (miscarriage) OR (clinical pregnancy rate) OR (livebirth rate) OR (preterm birth) OR (preterm labor) OR (preterm delivery) OR (premature labor) OR (premature delivery) OR (premature birth) OR (miscarriage) OR (stillbirth) OR (cesarean section) OR (c-section) OR (caesarean) OR (low birth weight) OR (small for gestational age) OR (placenta previa) OR (placenta praevia) OR (pre-eclampsia) OR (preeclampsia) OR (hypertensive disorders in pregnancy) OR (gestational hypertension) OR (pregnancy induced hypertension) OR (postpartum hemorrhage) OR (spontaneous hemoperitoneum) OR (antepartum hemorrhage) OR (abruptio placentae) OR (placental abruption) OR (growth restriction) OR (fertilisation rate) OR (fertilization rate) OR (intestinal perforation) OR (bowel perforation) OR (cholestasis) OR (mid-trimester loss) OR (implantation rate) OR (ovarian response) OR (cycle cancellation))

**#3** All Text=((cohort) OR (prospective) OR (retrospective) OR (follow-up) OR (longitudinal))

**#4** #1 AND #2 AND #3
