# Supplementary material for: Association between adenomyosis and maternal and neonatal outcomes: a systematic review and meta-analysis
Source: Front Med (Lausanne). 2026 Mar 31;13:1772838. doi: 10.3389/fmed.2026.1772838 (PMC13076185; doi:10.3389/fmed.2026.1772838)

**FIGURE S1** Subgroup analysis of clinical pregnancy rate. (A) Subgroup=Caucasian; (B) Subgroup=Asian; (C) Subgroup=Mixed; (D) Subgroup=Assisted reproductive technology.


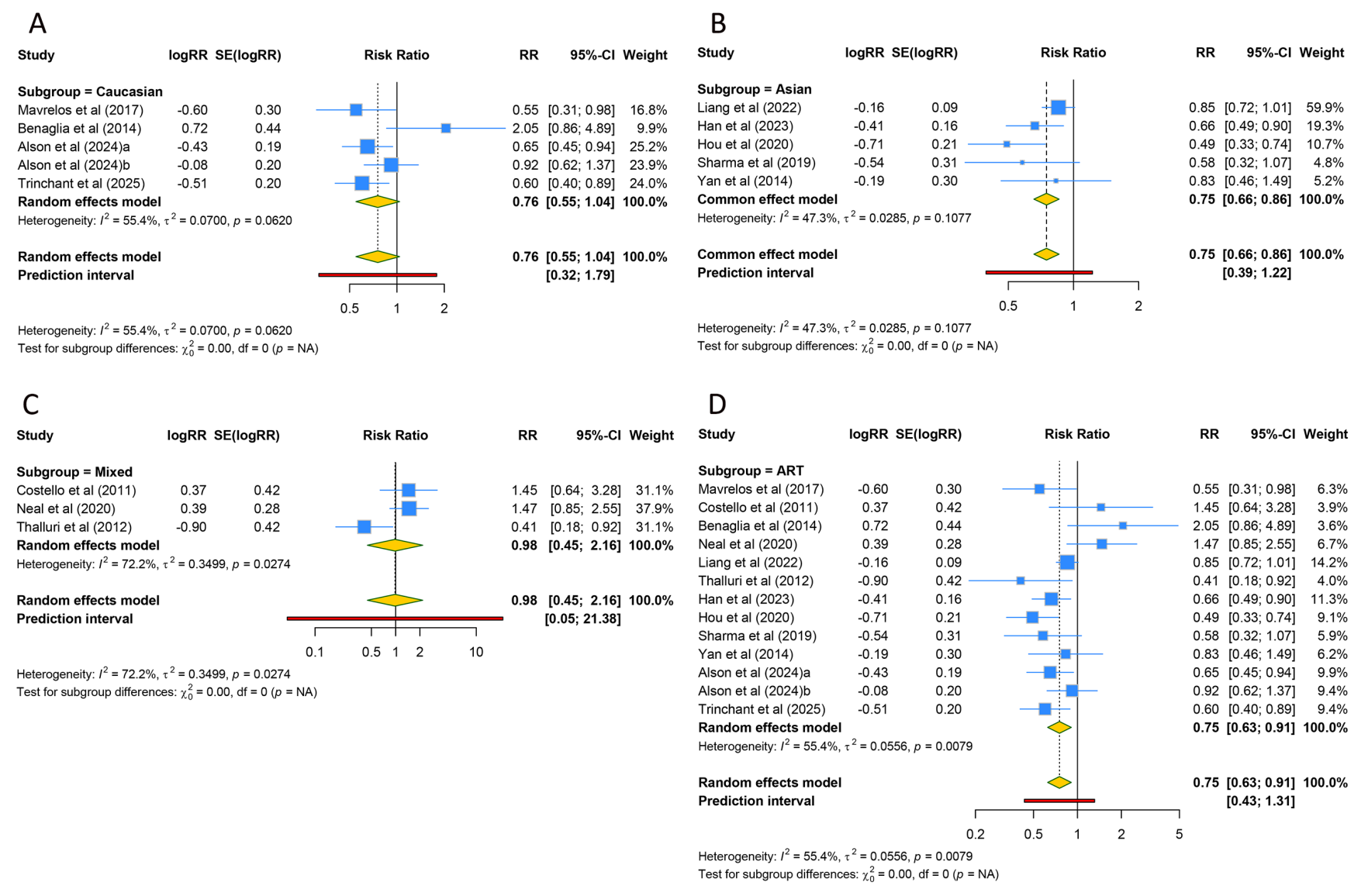


**FIGURE S2** Subgroup analysis of live birth rate. (A) Subgroup=Caucasian; (B) Subgroup=Asian; (C) Subgroup=Mixed; (D) Subgroup=Assisted reproductive technology.


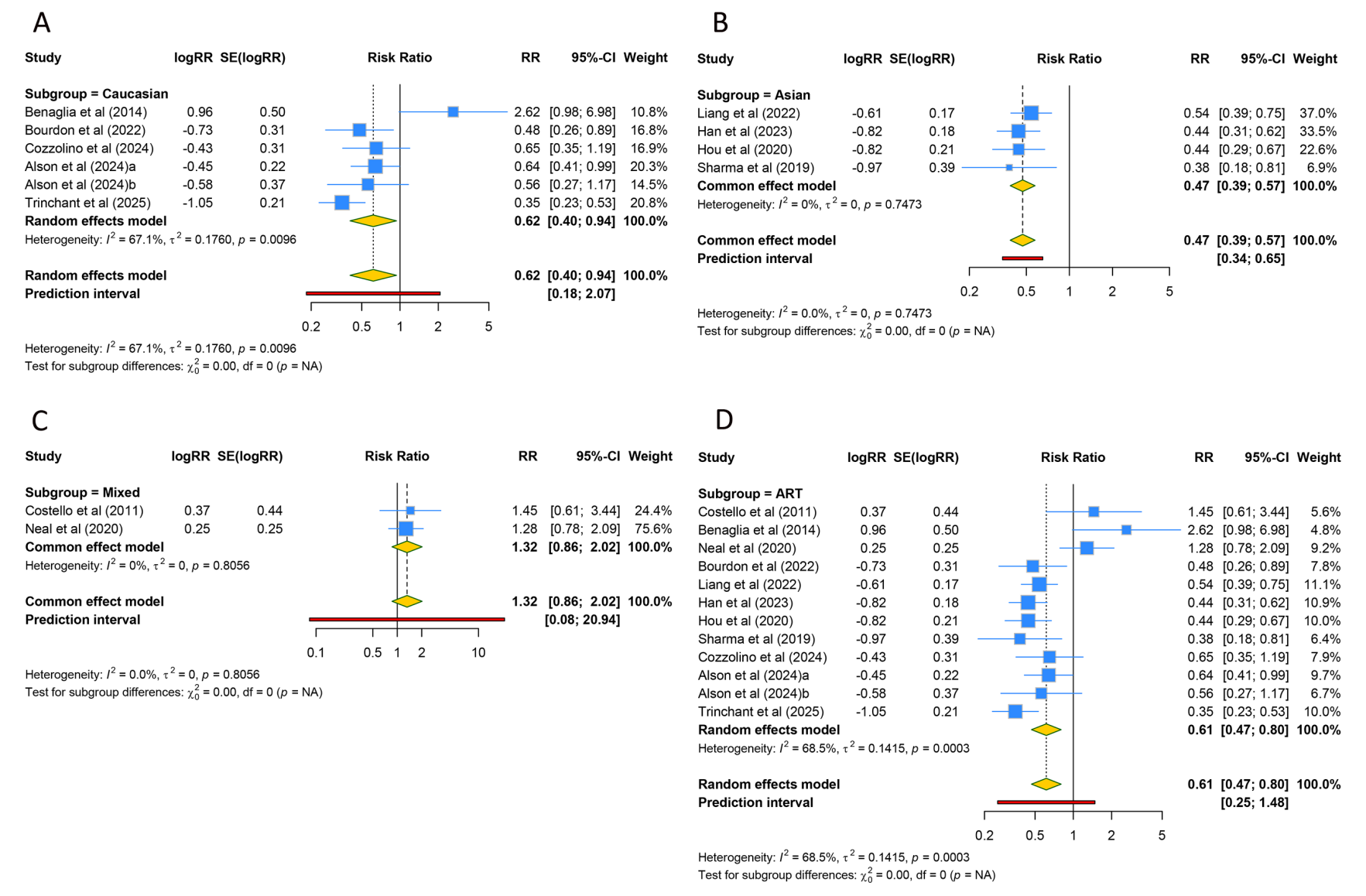


**FIGURE S3** Subgroup analysis of implantation rate. (A) Subgroup=Caucasian; (B) Subgroup=Asian; (C) Subgroup=Mixed; (D) Subgroup=Assisted reproductive technology.


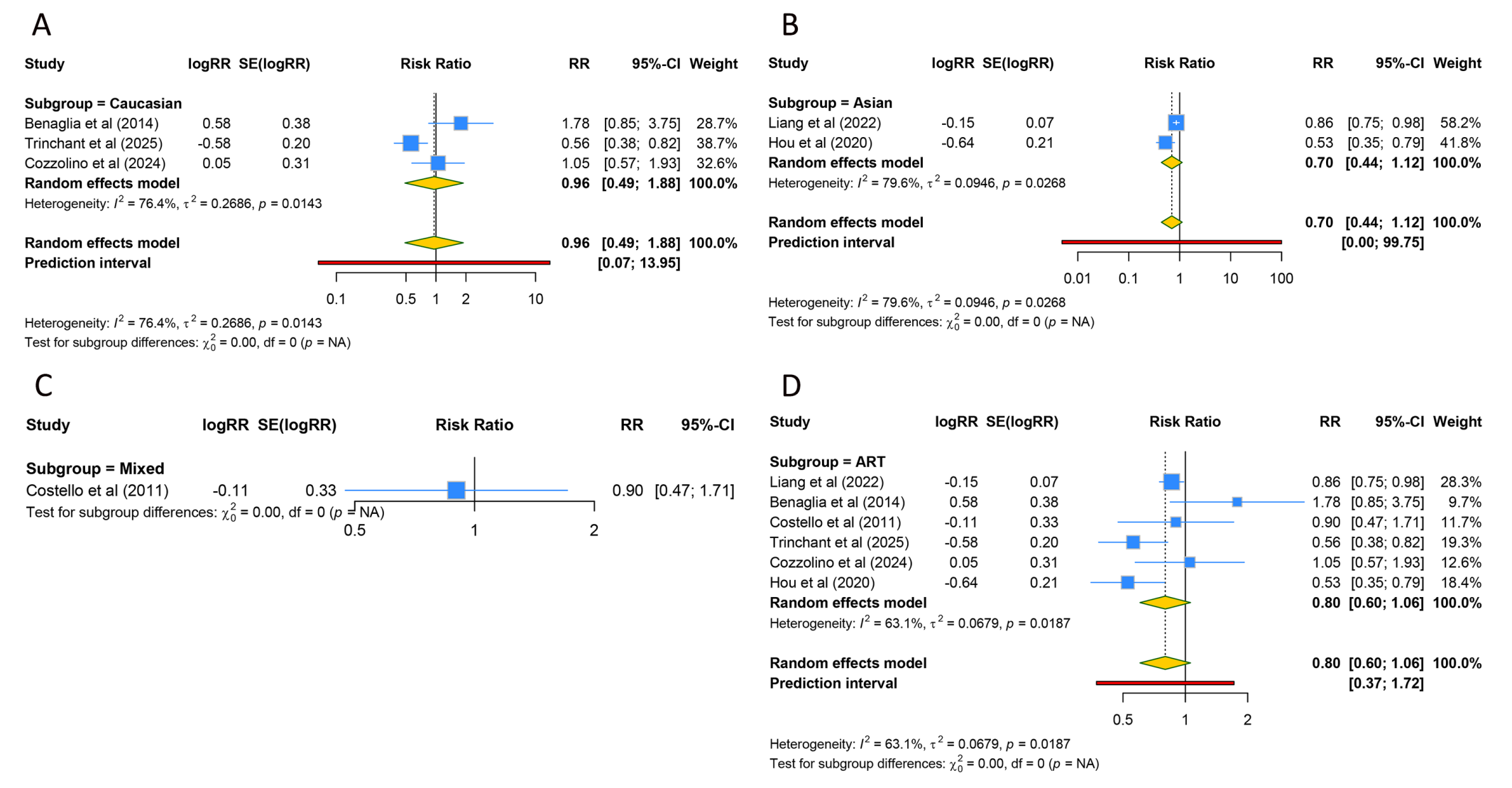


**FIGURE S4** Subgroup analysis of miscarriage. (A) Subgroup=Caucasian; (B) Subgroup=Asian; (C) Subgroup=Mixed; (D) Subgroup=Assisted reproductive technology (ART); (E) Subgroup=ART and natural conception.


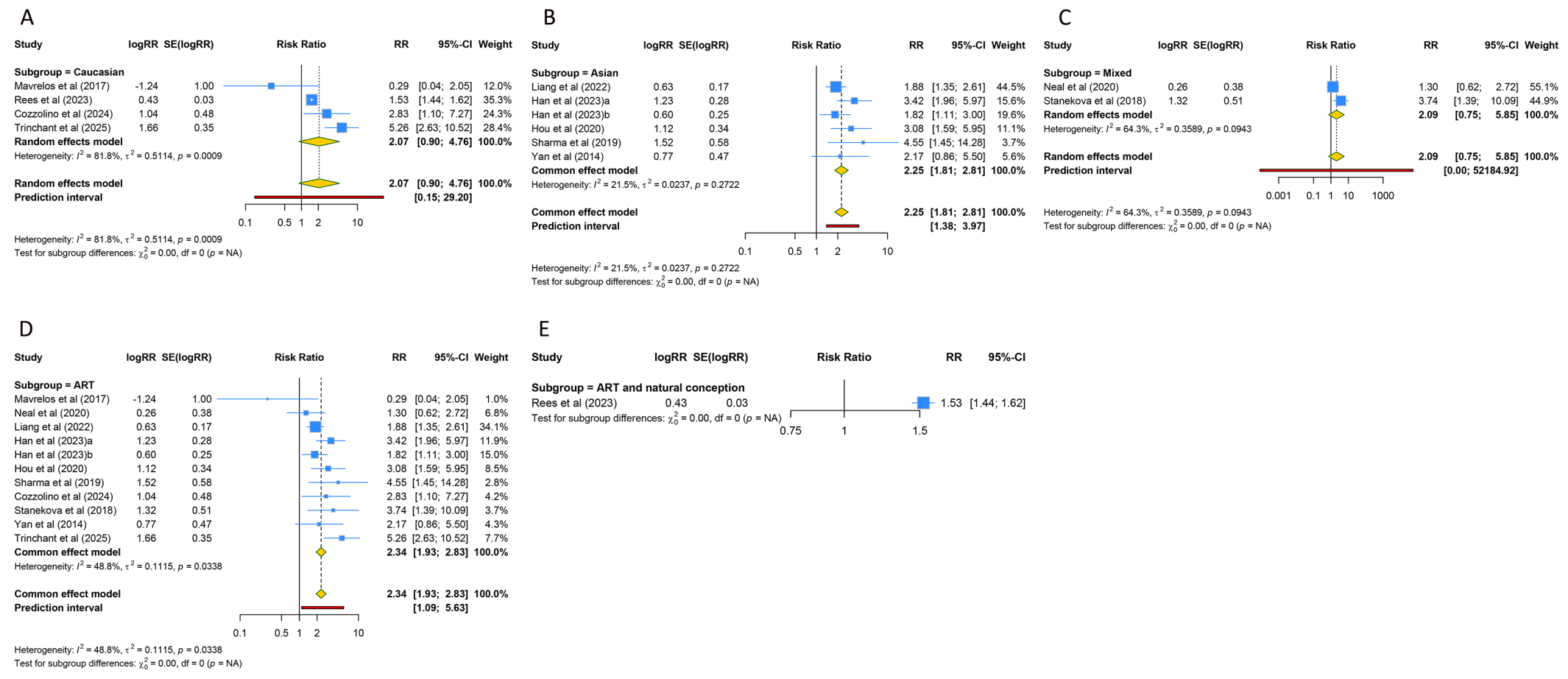


**FIGURE S5** Subgroup analysis of preterm birth. (A) Subgroup=Caucasian; (B) Subgroup=Asian; (C) Subgroup=Mixed; (D) Subgroup=Assisted reproductive technology (ART); (E) Subgroup=ART and natural conception.


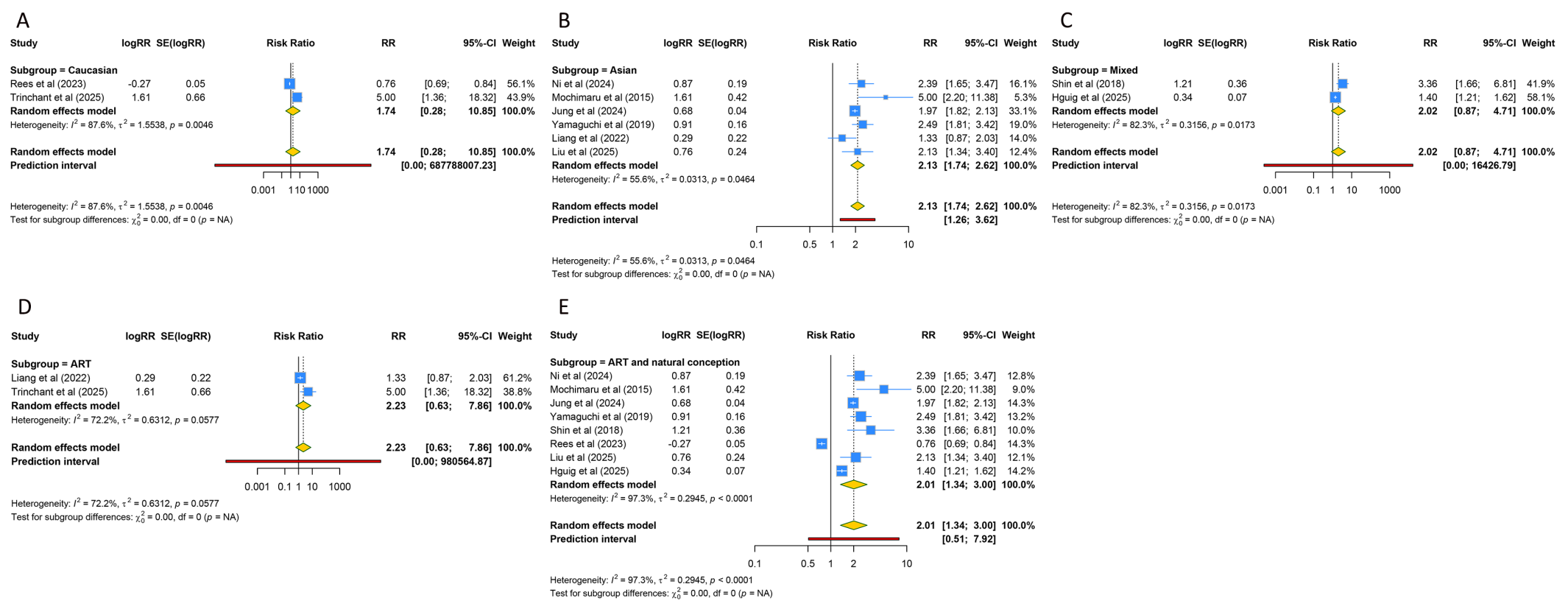


**FIGURE S6** Subgroup analysis of small for gestational age. (A) Subgroup=Caucasian; (B) Subgroup=Asian; (C) Subgroup=Assisted reproductive technology and natural conception.


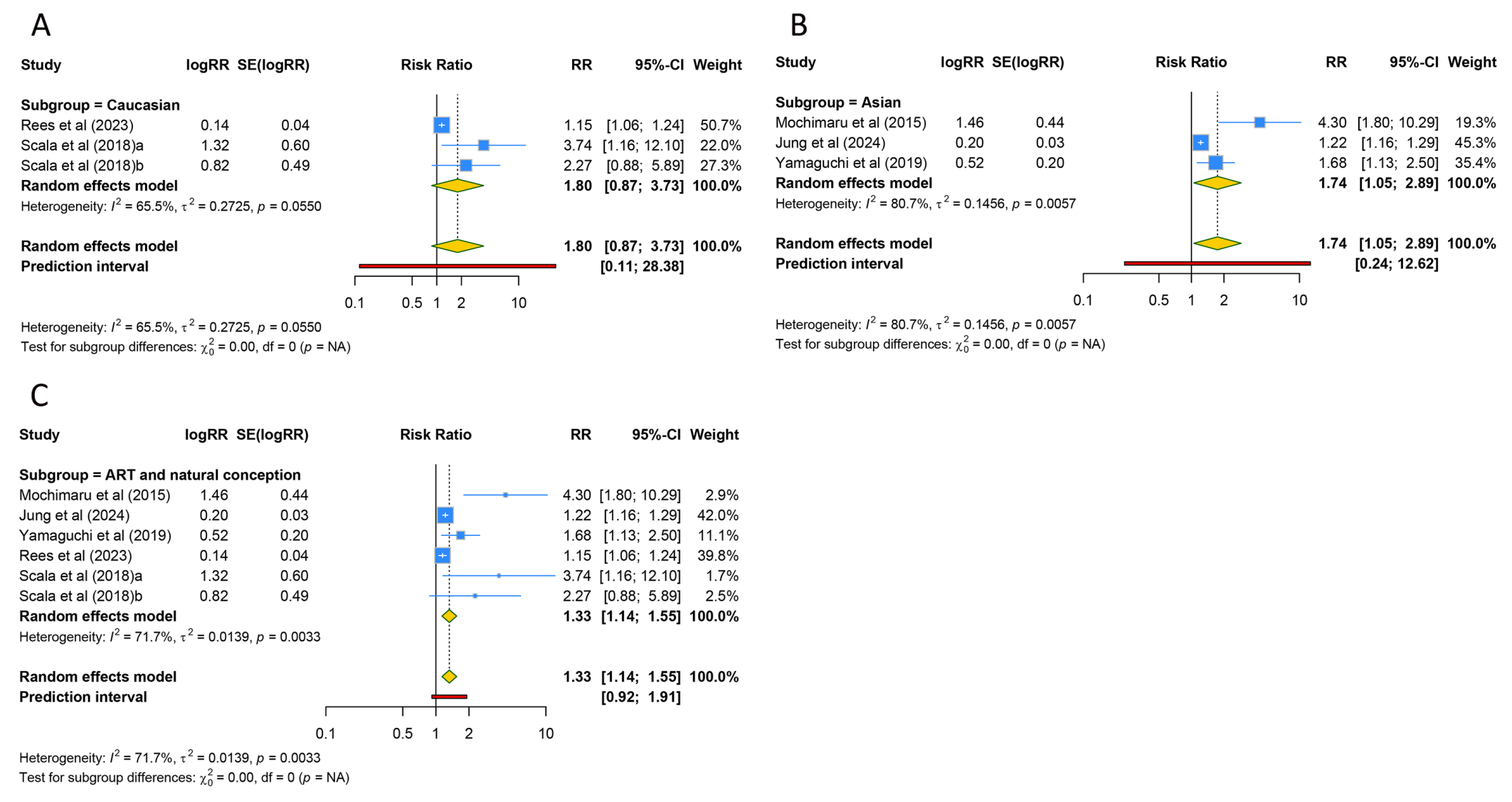


**FIGURE S7** Subgroup analysis of placenta previa. (A) Subgroup=Caucasian; (B) Subgroup=Asian; (C) Subgroup=Assisted reproductive technology (ART); (D) Subgroup=ART and natural conception.


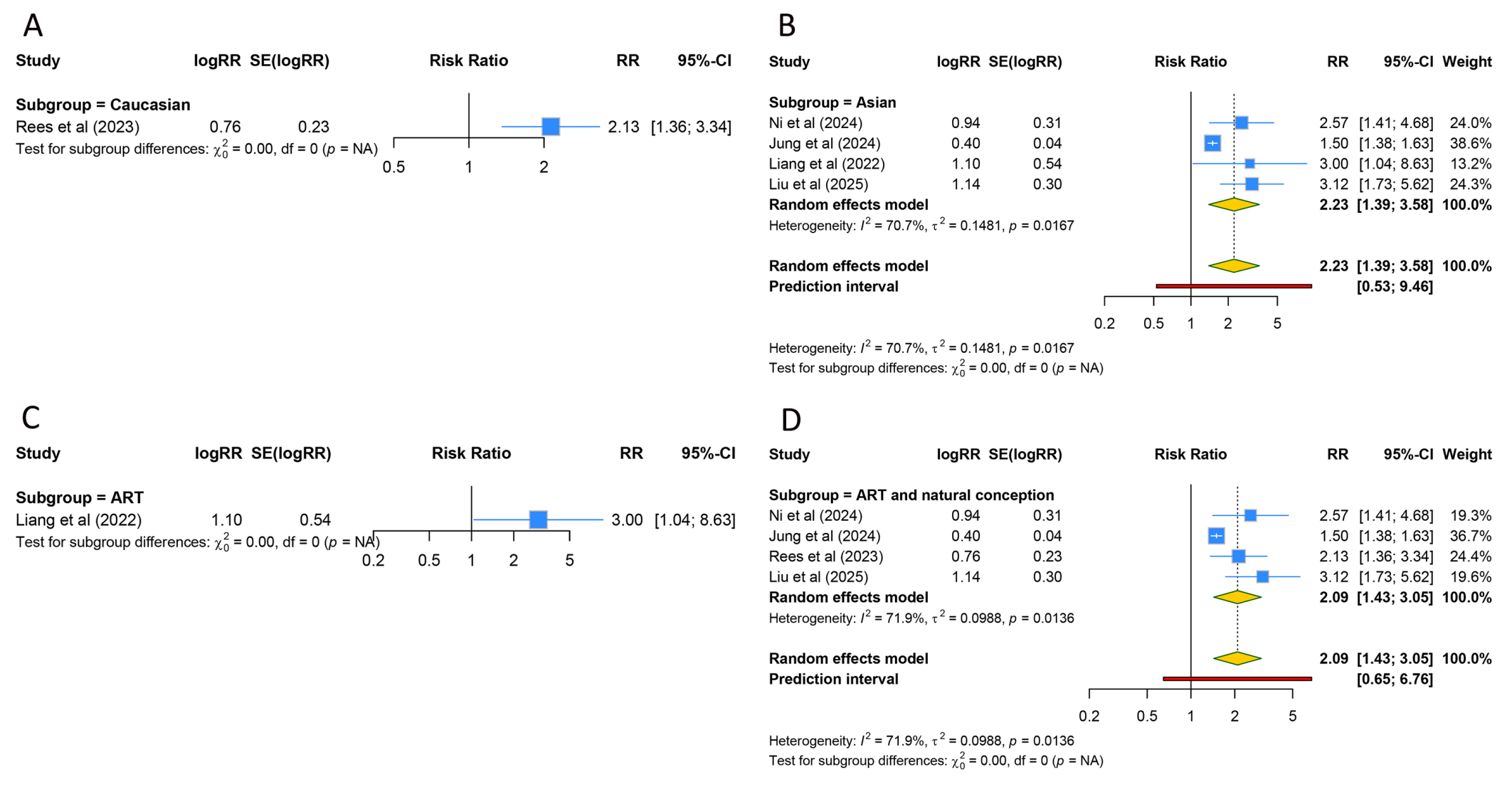


**FIGURE S8** Subgroup analysis of cesarean section. (A) Subgroup=Caucasian; (B) Subgroup=Asian; (C) Subgroup=Assisted reproductive technology (ART); (D) Subgroup=ART and natural conception.


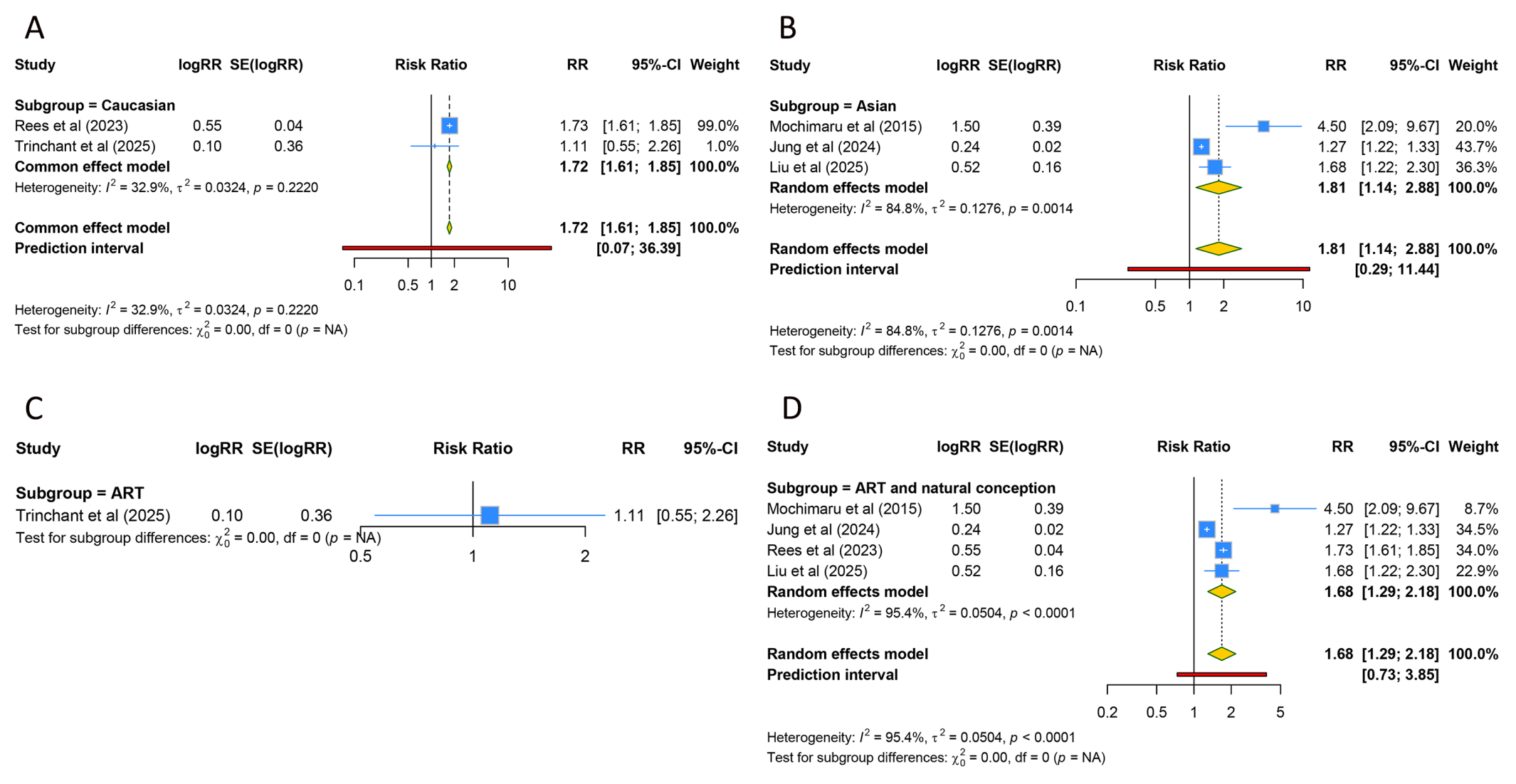


**FIGURE S9** Subgroup analysis of low birth weight. (A) Subgroup=Caucasian; (B) Subgroup=Asian; (C) Subgroup=Mixed; (D) Subgroup=Assisted reproductive technology (ART); (E) Subgroup=ART and natural conception.


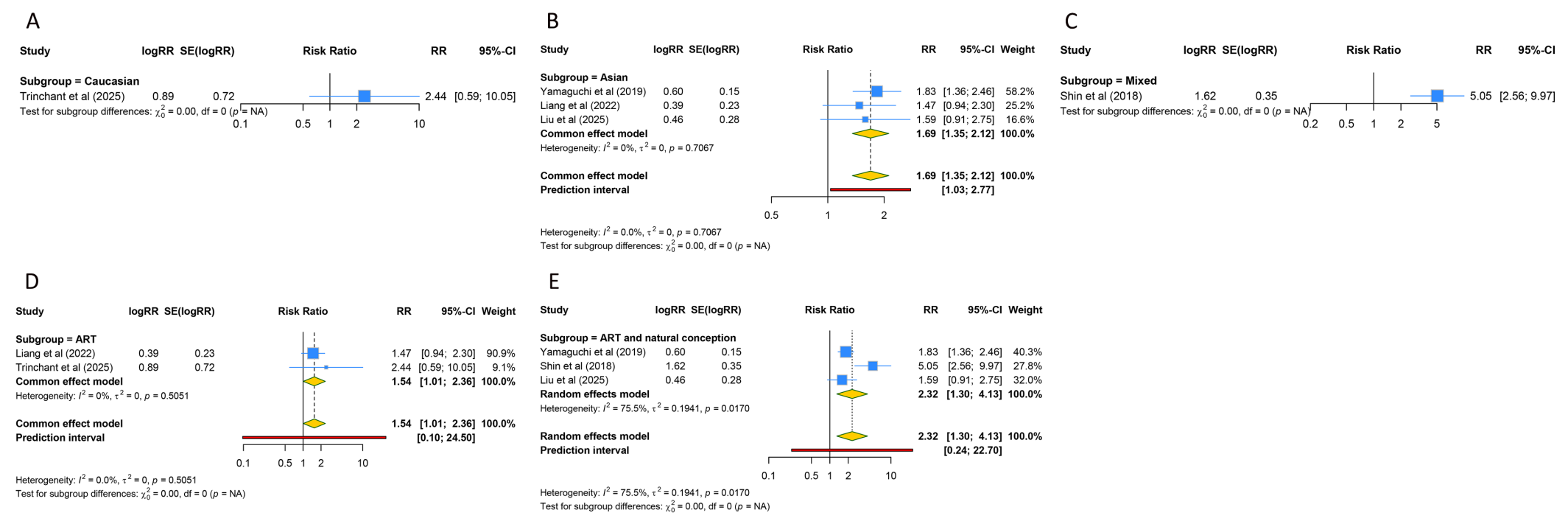


**FIGURE S10** Subgroup analysis of pre-eclampsia. (A) Subgroup=Asian; (B) Subgroup=Mixed; (C) Subgroup=Assisted reproductive technology (ART); (D) Subgroup=ART and natural conception.


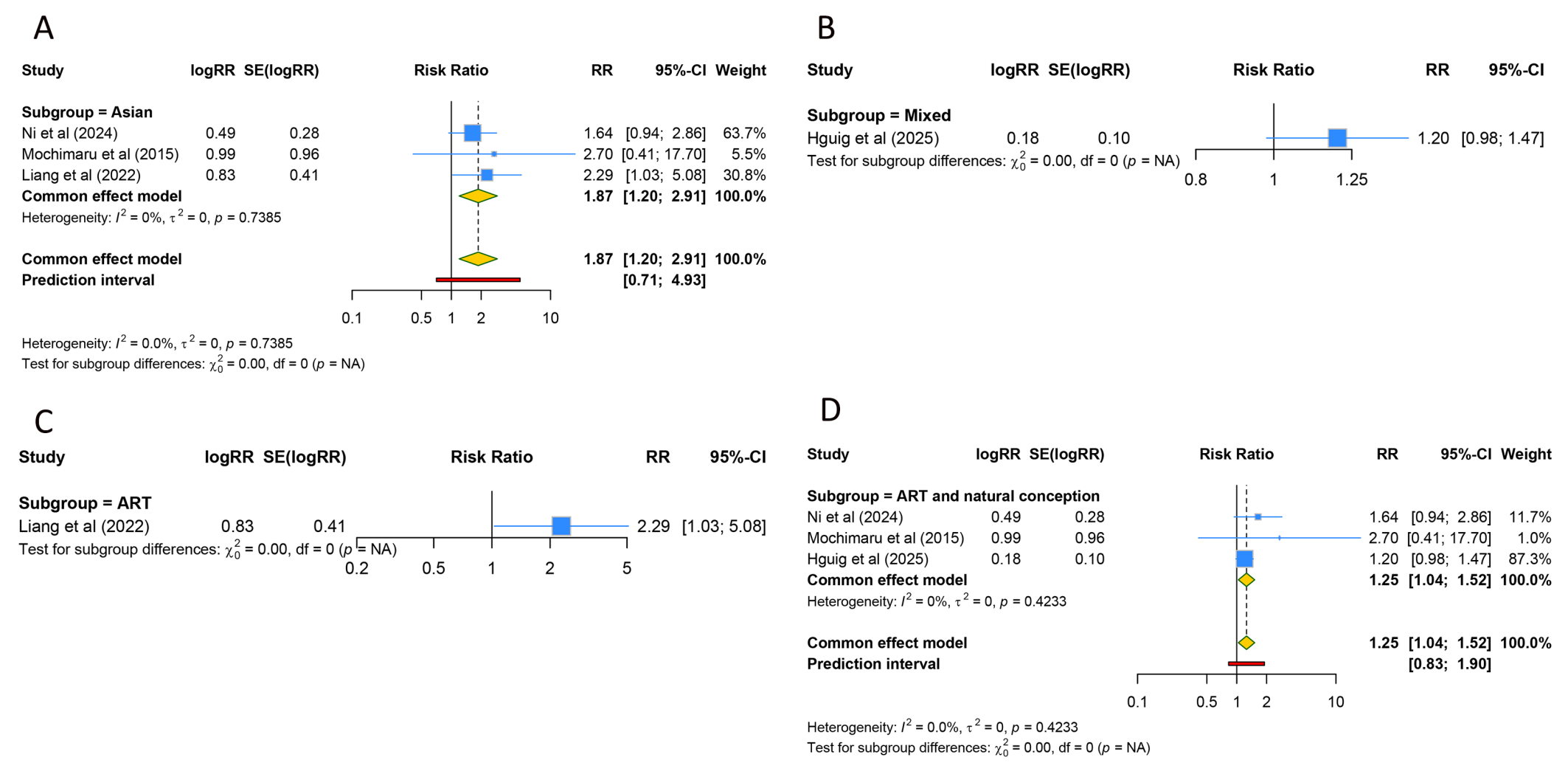


**FIGURE S11** Subgroup analysis of postpartum hemorrhage. (A) Subgroup=Caucasian; (B) Subgroup=Asian; (C) Subgroup=Mixed; (D) Subgroup=Assisted reproductive technology and natural conception.


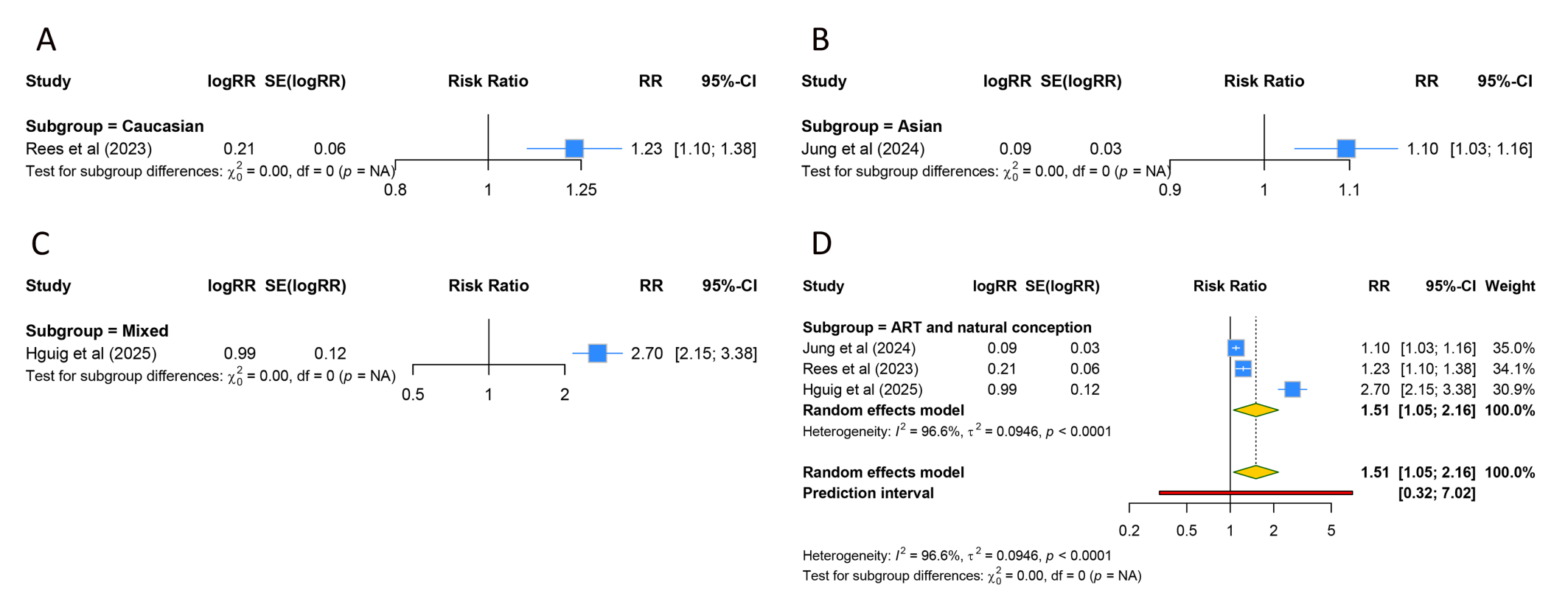


**FIGURE S12** Subgroup analysis of hypertensive disorders of pregnancy. (A) Subgroup=Caucasian; (B) Subgroup=Asian; (C) Subgroup=Mixed; (D) Subgroup=Natural conception; (E) Subgroup=Assisted reproductive technology and natural conception.


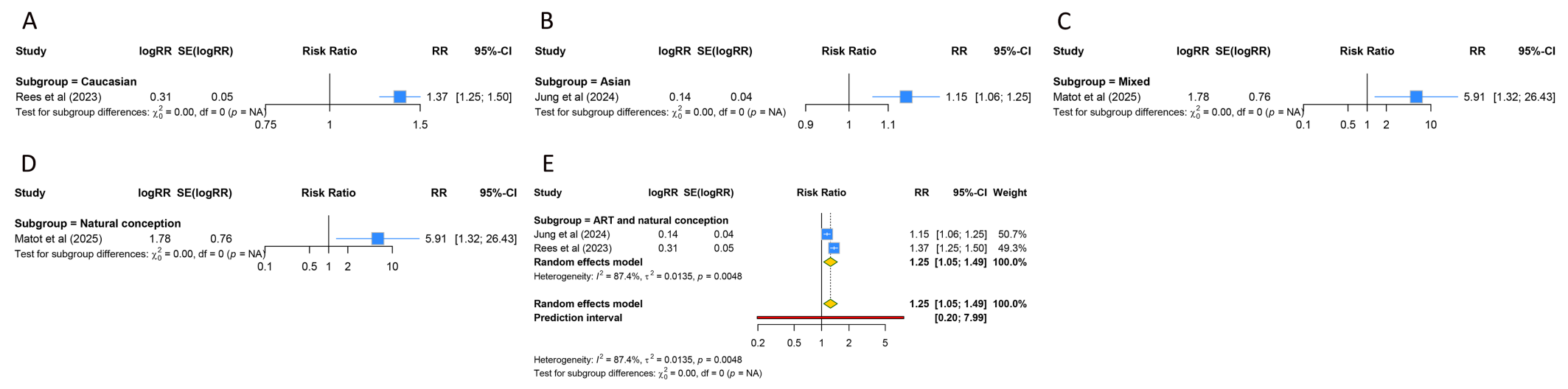


**FIGURE S13** Subgroup analysis of preterm premature rupture of membranes. (A) Subgroup=Caucasian; (B) Subgroup=Asian; (C) Subgroup=Mixed; (D) Subgroup=Assisted reproductive technology and natural conception.


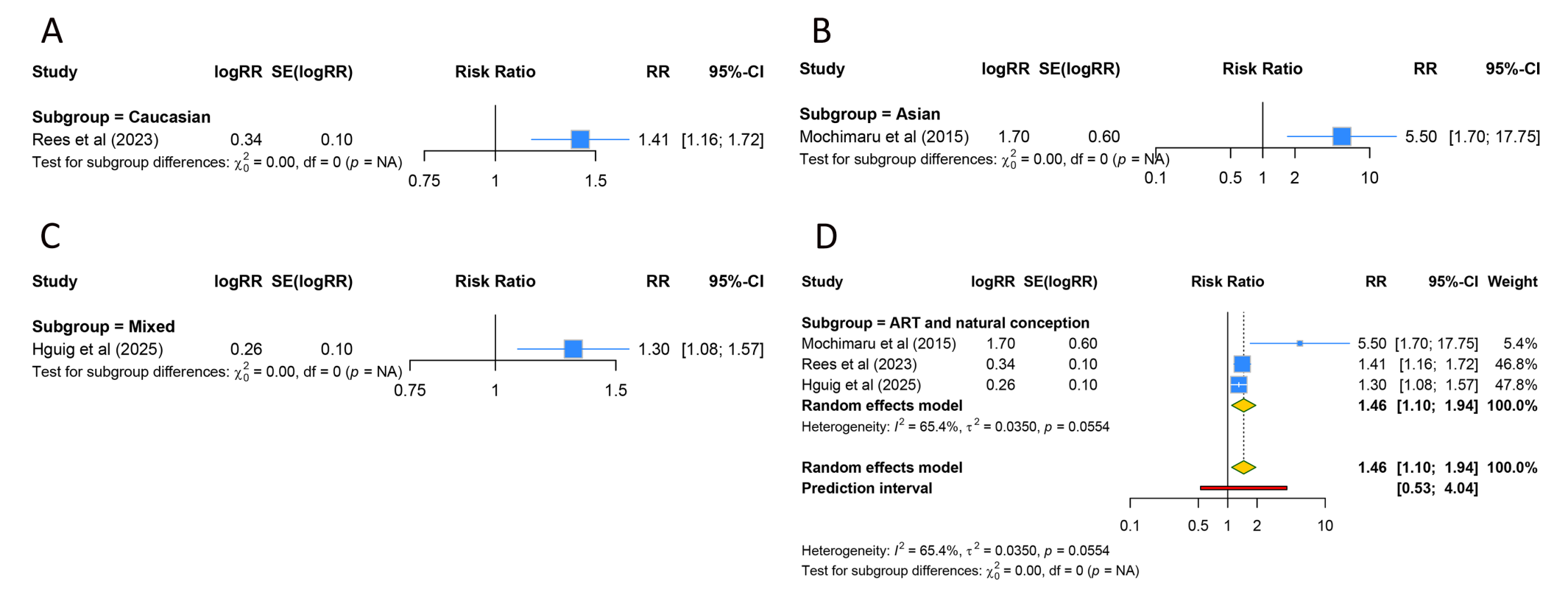


**FIGURE S14** Sensitivity analysis for the pooled results of (A) clinical pregnancy rate, (B) live birth rate, (C) miscarriage, and (D) preterm birth.


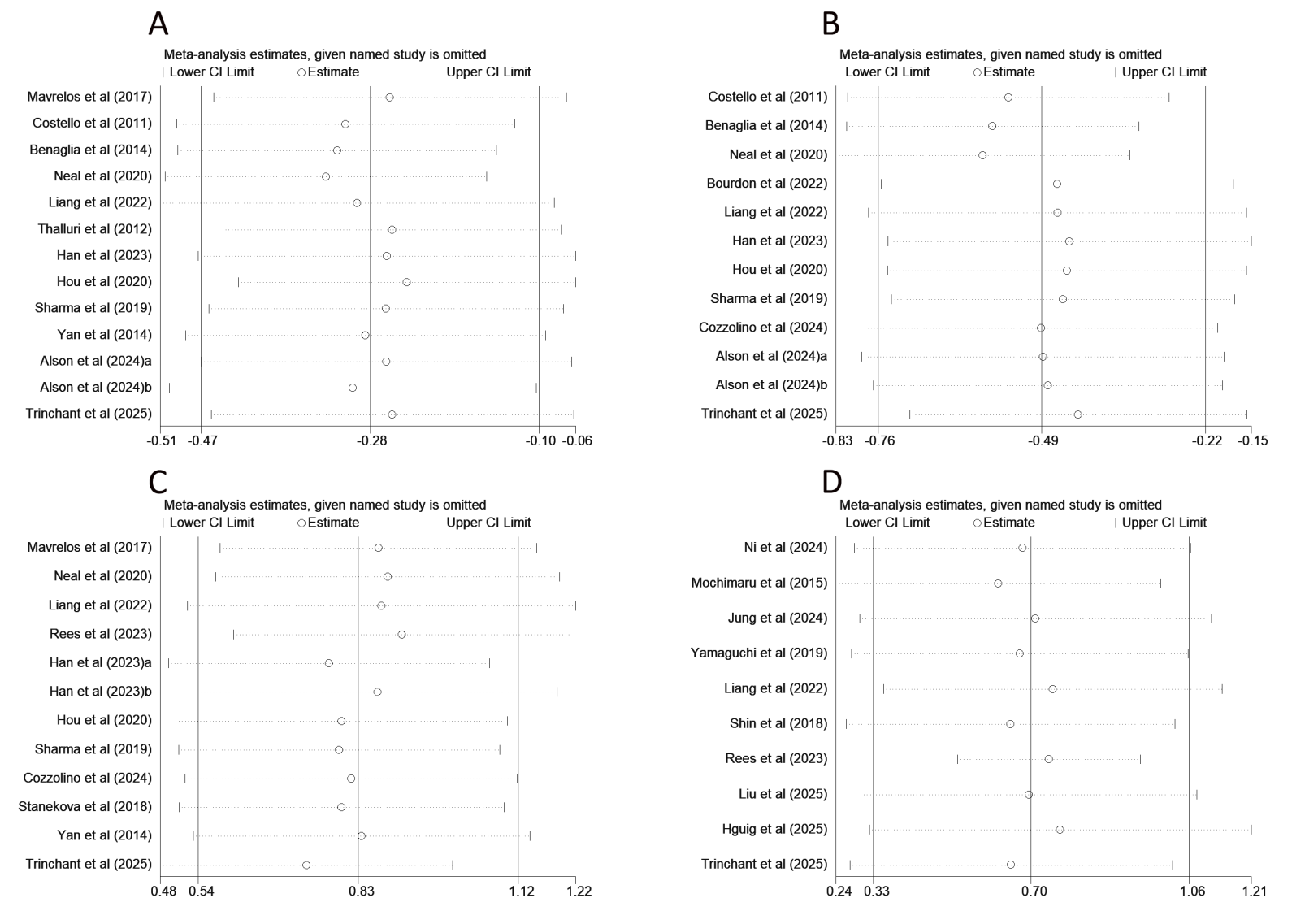


**FIGURE S15** Funnel plots of the pooled results of (A) clinical pregnancy rate, (B) live birth rate, (C) miscarriage, and (D) preterm birth.


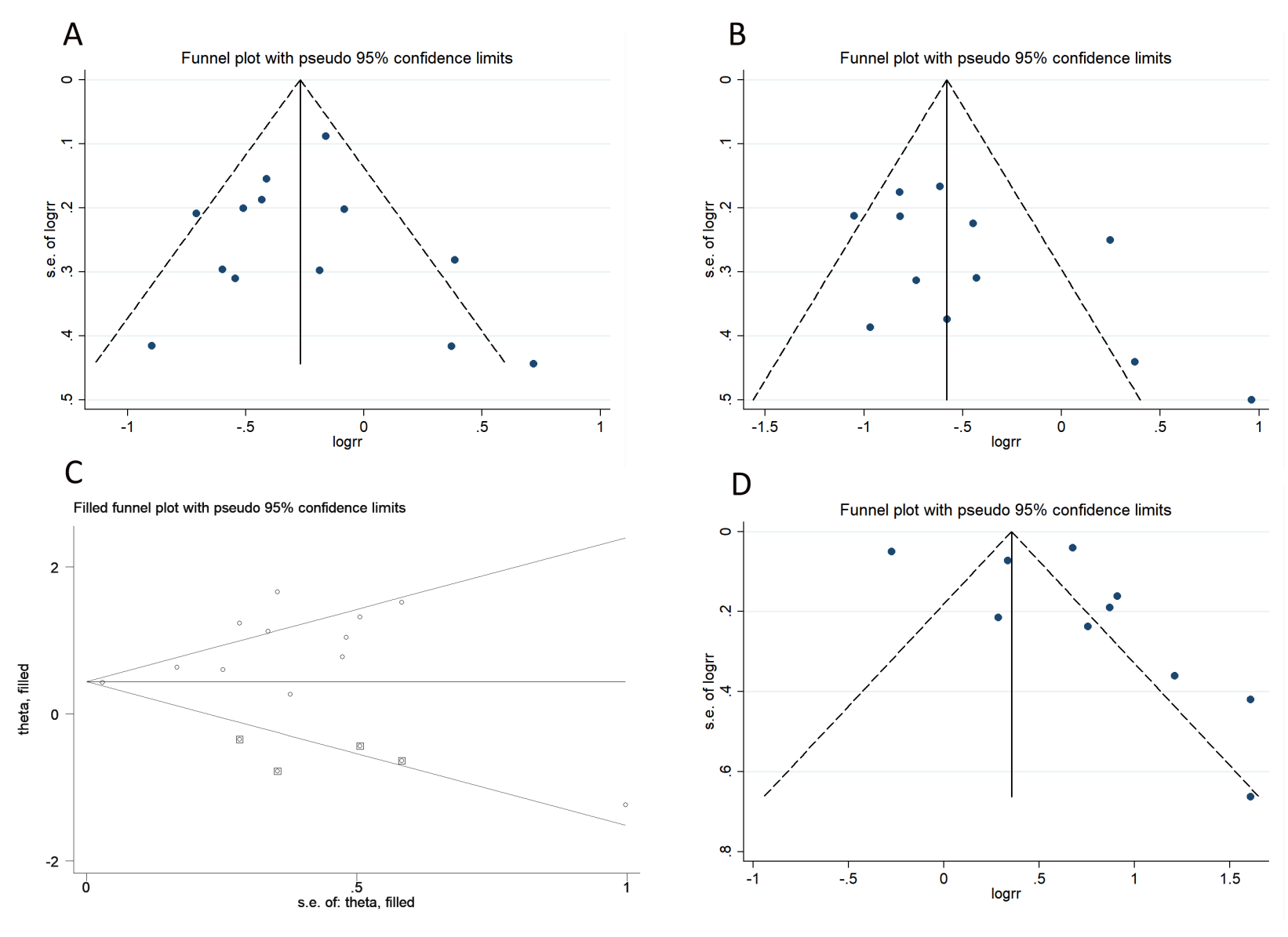

Supplement: Supplementary file 3 [file Data_Sheet_2.docx]
